# Effects of gear modifications in a North Atlantic pelagic longline fishery: A multiyear study

**DOI:** 10.1371/journal.pone.0292727

**Published:** 2023-10-19

**Authors:** Françoise D. Lima, Hugo Parra, Rita B. Alves, Marco A. R. Santos, Karen A. Bjorndal, Alan B. Bolten, Frederic Vandeperre

**Affiliations:** 1 IMAR–Institute of Marine Research, Departamento de Oceanografia e Pescas, Universidade dos Açores, Horta, Portugal; 2 Institute of Marine Sciences—Okeanos,–Departamento de Oceanografia e Pescas, Universidade dos Açores, Horta, Portugal; 3 ISPA—Instituto Universitário de Ciências Psicológicas, Sociais e da Vida, Lisboa, Portugal; 4 DROTRH–Direção Regional do Ordenamento do Território e dos Recursos Hídricos, Governo dos Açores, Horta, Portugal; 5 ACCSTR–Archie Carr Center for Sea Turtle Research and Department of Biology, University of Florida, Gainesville, FL, United States of America; Instituto Portugues do Mar e da Atmosfera, PORTUGAL

## Abstract

The threat of population declines caused by pelagic longline fisheries in the Atlantic has increased the concern to find strategies that minimize the bycatch and mortality of non-target marine animals. Gear modification, such as the use of circle hooks instead of conventional J-hooks, has been identified as an effective bycatch reduction strategy in different pelagic longline fisheries around the world. This study aimed to verify the effectiveness of the use of circle hooks by quantifying catch rates, relative size selectivity, and anatomical hooking position for the most common target species (swordfish, *Xiphias gladius*, and blue shark, *Prionace glauca*), and some bycatch species (loggerhead sea turtles, *Caretta caretta*, and shortfin mako, *Isurus oxyrinchus*) caught by the Azorean longline fishing fleet. The trial was conducted for five consecutive years (2000–2004) using eight different types of hooks. In general, the blue shark catches using circle hooks were significantly higher compared to J (Mustad 9/0). The circle hooks also showed high probabilities of catching juvenile blue sharks. Conversely, the circle hooks were efficient in reducing the loggerhead sea turtle bycatch and were related to fewer catches of small sea turtle individuals. The use of circle hooks was also associated with reduced swordfish catches compared to J (Mustad 9/0), and the effect of hook types on length at capture was only significant for Circle (L. & P. 18/0—CLP18) and Ringed Tuna (RT). No significant differences were observed comparing hook type to either catch rates or size selectivity for shortfin mako. Additionally, circle hooks were more likely to lodge in the mouth than in deeper anatomical positions, when compared to J (Mustad 9/0), for the four species analysed. The present study demonstrated that the use of circle hooks could mitigate the impact of the pelagic longline fisheries in the Azores by decreasing the bycatch of sea turtles and reducing animal injuries caused by deep hooking.

## Introduction

Global fisheries bycatch is one of the major threats to the world’s marine biodiversity, with annual average total discards around 7.3 million t [1, 2]. The pelagic longline fisheries commonly target large predatory fishes, such as swordfish (*Xiphias gladius*, Linnaeus 1758), tunas, commom dolphinfish (*Coryphaena hippurus* Linnaeus, 1758), and blue sharks (*Prionace glauca* Linnaeus, 1758) [3, 4]. Several non-target species with K-selected life strategies and conservation concerns (sharks, sea turtles, seabirds, marine mammals) become incidentally entangled or hooked and are frequently discarded for regulatory or economic reasons [5, 6].

The pelagic longline fishing activity in Northeast Atlantic demonstrates a distinct seasonality, characterized by a predominance of swordfish captures during the autumn, primarily concentrated west of mainland Portugal. Throughout the spring and summer seasons, the fishing effort disperses westward toward the Azores, with vessels primarily targeting blue shark [7]. Incidental captures are characterized especially by shortfin mako (*Isurus oxyrinchus* Rafinesque, 1810), bigeye thresher (*Alopias superciliosus* Lowe, 1841), loggerhead sea turtles (*Caretta caretta* Linnaeus, 1758), among others [8, 9].

The fleet operating in the Azores is composed of a small regional fleet and long-distance vessels from Spain and mainland Portugal (Fauconnet et al. 2019). This fishery catches an annual average of 1,310 t of blue sharks, 290 t of swordfish, and about 82 t of shortfin mako, landing mostly outside of the archipelago [8, 10, 11]. In addition, an estimated 107 t of sea turtles are incidentally captured each year by the pelagic longline fishery in the region [12].

During their last stock assessments, the North Atlantic blue shark and swordfish stocks, respectively in 2015 and 2022, were not considered to be overfished or subject to overfishing. Those species are managed by the International Commission for the Conservation of Atlantic Tunas (ICCAT) by establishing annual Total Allowable Catches (TAC), currently of 39,102 t and 13,200 t for blue shark [13] and swordfish [14], respectively, to ensure sustainable exploitation. The Standing Committee on Research and Statistics (SCRS), the scientific advisory body of ICCAT, warns that the fishery status of blue sharks should be interpreted carefully due to uncertainties in the stock assessments. In the case of shortfin mako stock in the North Atlantic, since this species has vulnerable biological characteristics, the SCRS of ICCAT recommends a non-retention policy with zero TAC [15], which recently entered into force in the European Union (Council Regulation 2022/109), but it was already implemented in the Azores since 2019 [16]. According to the IUCN, three of the four most caught species in the Azorean pelagic longline fishery are under some level of conservation concern, as the blue shark populations are near threatened [17], the loggerhead turtle is vulnerable [18], and the shortfin mako is endangered [19].

The threat of population decline caused by the impact of longline fisheries has increased the interest of scientists, fisheries companies, and government agencies in finding strategies that minimize the incidental capture and mortality of non-target marine animals [20, 21]. However, effective bycatch mitigation measures are often complex because they must balance ecological and economic benefits [22]. A combination of hook design, type of bait, depth, gear specifications, gear soak duration, and alteration of the temporal and spatial aspects of fishing are examples of practices that can be modified to reduce incidental catches [23]. Gear modifications to increase selectivity are expected to be one of the most effective tools in reducing bycatch mortality [21, 24]. Several studies have demonstrated the use of circle hooks as an effective bycatch reduction strategy since they are commonly associated with lower rates of deep-hooking and higher survival rates at gear haulback, as well as increased catch of some target species [23, 25, 26]. Circle hooks differ from conventional J-hooks as the point of the gear is oriented perpendicular to the shank. This rounded shape allows the hook to slide over soft tissue in the mouth and oesophagus and rotate as the hook exits the mouth of a fish, which facilitates jaw hooking [20, 27].

Considering that circle hooks have been associated with significant decreases in injury and mortality rates of bycatch species, it has been mandatory for all ICCAT fleets since 2022 to either employ circle hooks or utilize finfish bait. Furthermore, the use of circle hooks is obligatory in the US longline Atlantic fishery [3, 28]. Since January 2020, the pelagic longline fleet operating in the Azores is required to use straight circle hooks with a gap of at least 30 mm [29]. However, incorporation of the permitted hooks has been gradual, allowing the fleet sufficient time to adapt to the new fishing gear.

Although circle hooks are generally recommended to reduce bycatch rates, some studies comparing these hooks to traditional ones (Ringed Tuna, J-hooks) used in pelagic longline fisheries have generated variable results [4, 25, 27, 30]. A compilation of studies on hook effectiveness for 43 species [20] found that catch rates with circle hooks vs. J-hooks were higher for shark and tuna species, lower for sea turtles and other fishes, and mixed for the billfish species. On the other hand, [31] conducted a meta-analysis that included 15 studies and concluded that the circles hook had no significant quantitative effects on the blue shark catches. Due to the lack of convergent effects of circle and traditional hooks on target and non-target species, the wider introduction of circle hooks in commercial fisheries is recommended only when consistent scientific data from field trials prove its effectiveness [32]. The effectiveness of specific types and sizes of circle hooks should be assessed and tested within each fishery before adopting them as management and conservation measures [24, 32].

This study reports the effectiveness of circle hooks compared to conventional hooks in the pelagic longline fishery in the Azores. The aim was to quantify the relative effects of hook types on catch rates, relative size selectivity, and anatomical hooking position for the most common target and bycatch species (swordfish, blue shark, loggerhead sea turtle, and shortfin mako), using eight different types of hooks (circle, tuna and J-hooks).

## Material and methods

### Study area

**Fig 1:**
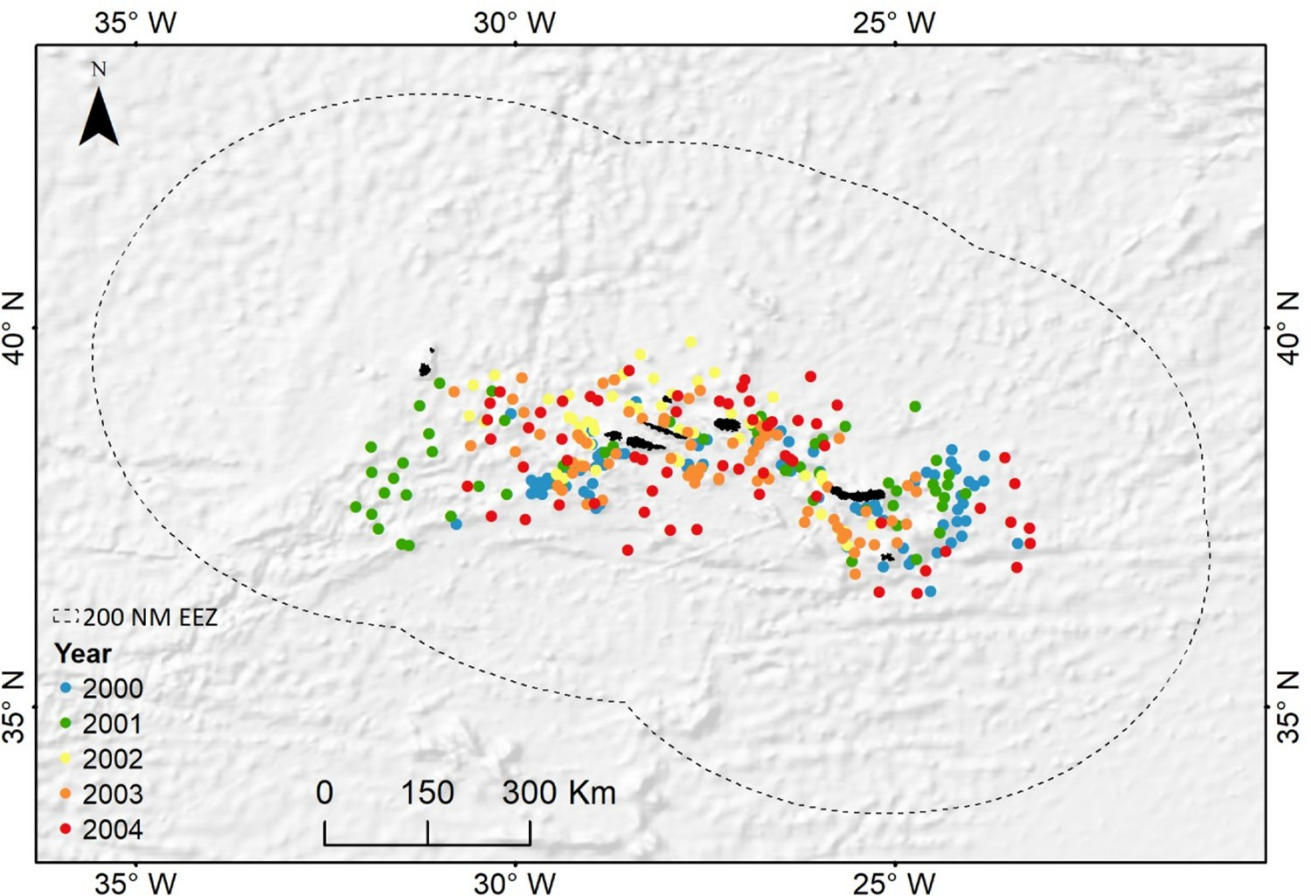
Map of the study area in the Azorean Exclusive Economic Zone (EEZ), with the centre locations of sets in each year represented by different colors. The map was build using ArcMap in ArcGIS Desktop 10.8.1 [40].

The Azores Archipelago, an autonomous region of Portugal, is a group of nine volcanic islands located on the Mid-Atlantic Ridge in the North Atlantic (Fig 1). The Azorean Exclusive Economic Zone (EEZ) covers an area located between 33° and 43° N, 20.5° and 30.5° W, where fishing occurs around the island slopes and several seamounts [10]. This area is considered an important foraging and developmental ground for loggerhead sea turtles originating mostly from the southeast U.S.A [33–37], a nursery area for blue sharks [38], and an essential habitat (hotspot) for key groups of vertebrate marine megafauna, mainly migratory species [39].

### Pelagic longline fishery trial

The Azores pelagic longline fishery trial was conducted for five consecutive years, from 2000 through 2004 (Table 1). The main goal was to examine the effects of terminal gear modification (hook type) on the bycatch rates and anatomical hooking position for the loggerhead sea turtle. Two fishery observers participated in the trials, with one observer present on the vessel at each fishing cruise. They collected and recorded the data according to the protocol of the Azores Fisheries Observation Program (POPA). Fishery sets were carried out during summer and autumn (July–December), periods of high abundance of sea turtles in the area [33]. Three different hooks were used in each year, except in 2004, when only two were tested. In total, eight different hooks of three types were tested (Circle, J-hooks, and Ringed Tuna) (Table 1).

**Table 1:** Summary of the pelagic longline fishery trial detailing the number of hooks, sets, gear, and the hook types used from 2000 through 2004.

Year	Total Sets	Total hooks	Set distance (Km)	Hook Type	Hook abbreviation	Number of hooks	Gear	
							Type	Leader
2000	93	138121	5656	Straight J (Mustad 9/0 - #76800)	JM9	46040	Spanish	Mono
				Offset J (Mustad 9/0 - #76801)	JM9O	46040	Spanish	Mono
				Circle (Mustad 16/0 - #39960)	CM16	46041	Spanish	Mono
2001	60	88150	3544	Straight J (Mustad 9/0 - #76800)	JM9	29383	Spanish	Mono
				Circle (Mustad 16/0 - #39960)	CM16	29383	Spanish	Mono
				Circle (Mustad 18/0 - #39960)	CM18	29384	Spanish	Mono
2002	48	75511	3988	Circle (Mustad 16/0 - #39960)	CM16	25170	American	Wire
				Offset Cir. (Mustad 16/0 - #39966)	CM16O	25170	American	Wire
				Offset Cir. (L. & P. 18/0—Lindgren-Pitman)	CLP18O	25171	American	Wire
2003	73	114417	7225	Circle (Mustad 16/0 - #39960)	CM16	51000	American	Wire
				Circle (L. & P. 18/0—Lindgren-Pitman)	CLP18	50600	American	Wire
				Ringed Tuna (3.6 mm (OPI023)	RT	12817	American	Wire
2004	69	81681	6361	Circle (L. & P. 18/0—Lindgren-Pitman)	CLP18	40840	American	Wire
				Offset Cir. (L. & P. 18/0—Lindgren-Pitman)	CLP18O	40841	American	Wire

During the first two years (2000 and 2001), the trial was performed on the commercial fishing vessel “Mestre Bobicha”, 25.4 m long, using “Spanish-style” pelagic longline, which consists of a multifilament mainline (4 mm diameter) and fixed hook positions. This equipment used included weighted monofilament branchlines (1.8 mm) and nylon leaders. Eight hooks were deployed between buoys with branchlines ranging from 5.5 to 11 m, depending on sea conditions. The distance between the branchlines was 45 meters. The arrangement aimed to ensure standardization of soak time and hook depth by individually alternating the three hook types (A, B, C) used each year along the set (A, B, C, A, B, C,…). A large radar buoy (RB) was placed at the beginning of each set, followed by four small buoys (b) and one large buoy (B), according to the configuration described by [41].

In the following three years (2002, 2003, and 2004), the trial was conducted on the commercial fishing vessel “Mizar”, 24.5 m long. This vessel was equipped with an “American-style” longline gear featuring a monofilament mainline with a diameter of 3.5 mm. The branchlines consisted of monofilament (2.1 mm diameter) with wire leaders. There were four branchlines (16 m long) between buoys and approximately 80 meters of mainline between each branchline. The buoy lines were 6–24 m long, depending on sea conditions. The three hook types used each year were also individually alternated along the set (A, B, C, A, B, C,…) with four hooks between the buoys. Since the hooks were distributed alternately on the mainline, all hook types were evenly distributed in all possible positions between two floats.

Both types of gear were set one or two hours before sunset and retrieval began before sunrise. Captains on both vessels were told to fish as they normally would, other than using the hook distribution as mandated in the trial design. In total, 343 sets and 497,880 hooks (data analysed: 331 sets and 482,205 hooks) were deployed during this study (S1 Table in S1 File). The number of hooks per set varied between 632 and 1892 (mean = 1456.8 ± 187.4). The bait used in all fishing sets was squid.

Morphometric measurements of the fishes were taken with callipers to the nearest centimeter. For blue shark and shortfin mako, the pre-caudal length (PCL) was adopted as the primary measure for body size, while lower jaw fork length (LJFL) was used for swordfish. Minimum curved carapace length (CCL) was the standard length for sea turtles and was measured with a flexible measuring tape.

### Environmental variables

Environmental conditions are expected to influence the abundance and catch rates of target and non-target species in longline fisheries [41]. Therefore, some environmental variables were included in the analyses in order to understand their influence on catch rates and obtain more accurate estimates for the coefficients of the hook types: sea surface temperature (SST), sea surface height anomalies (SSHa), Chlorophyll, and lunar cycle. To avoid missing values due to cloud cover, we used weekly Reynolds Optimum Interpolation SST (NOAA Optimum Interpolation (OI) Sea Surface Temperature (SST) V2), a product calculated based on in situ and satellite SSTs and provided on a 1° grid. These SST data go back to 1993, covering the period in which this pelagic longline trial was carried out. For SSHa, we used the daily Delayed Time Sea Level Anomalies provided by AVISO. These altimetry data are a merged product from various altimetric missions (Topex/Poseidon, ERS-1/2, Jason-1, Envisat and OSTM/Jason-2) provided on a Mercator grid with a resolution of 1/3° × 1/3°. The lunar cycle was calculated from the illuminated fraction of the moon provided by the U.S. Naval Observatory [42] to obtain a variable ranging between −1 and 1, both representing the new moon, and 0 representing the full moon. The chlorophyll data were available from ACRI-ST, based on the Copernicus-GlobColour processor, for the Global Ocean Satellite Observations. The mean monthly values were retrieved for each year (2000–2004) in a spatial resolution of 4 km.

### Data analyses

The trial in the Azores EEZ caught 27,603 individuals of 34 species, including 19 bony fishes, 11 sharks, 3 turtles, and 1 bird species (S2 Table in S1 File). The analyses of gear modification effects on catch rates, size selectivity, and anatomical hooking position were conducted for the four most abundant species. It’s important to emphasize that when calculating catch rates, we are taking into account the retention of the animals on the line, as we did not have accurate information regarding bite-offs in this trial.

The blue shark was the predominant species captured in the fishery trial with 81.65% of total catches, followed by the swordfish (11.68%), loggerhead sea turtle (1.78%), and shortfin mako (1.39%) (S2 Table in S1 File). The summary of the analysed data, including number of individuals, catch per unit effort (CPUE—ind/1000 hooks), and size per species is showed in the Table 2.

**Table 2:** Summary of analysed data of the four most abundant species in the pelagic longline fishery trial in the Azorean EEZ.

Species		CPUE (ind/1000 hooks)			Size (cm)		
	NA	Mean	SD	Range	Mean	SD	Range
Blue shark	22410	42.21	46.87	0–224.78	121.43	33.24	51–290
Swordfish	3188	6.60	4.73	0–28.11	117.47	24.94	50–225
Loggerhead sea turtle	471	1.01	2.00	0–19.05	53.23	7.62	24.3–82.2
Shortfin mako	377	0.76	1.57	0–10.83	121.39	24.33	43–222

SD = standard deviation.

### Effect of the hook types on catch rates

We used Generalized Additive Mixed Models (GAMM) to test the effect of hook type and other variables on the catch rates of the four selected species. These models use non-parametric functions to fit non-linear relationships between the response and explanatory variables [43]. The models were run independently because the distribution of the response variable for each species was different, as were the species-specific relationships with explanatory variables, mainly environmental ones. The catch rates were modelled as species counts by hook type in each set, considering the influence of different variables. The effort (number of hooks) was used as offset and transformed in accordance with the link function used in the model family. The date was included as a random effect since it represents one set where we tested three hook types.

Exploratory analyses were performed to assess collinearity, outliers, zero inflation, and missing data of the variables for each model [44]. Data exploration revealed collinearity between SST and Chlorophyll, and the latter was removed from the final dataset in all models (S1 Fig in S1 File). The Leader Type variable was excluded from the analysis because it showed a high correlation with Year. Since Year provided a better fit to the models and allowed for improved resolution of the data, it was retained in the full models (S1 Table in S1 File). The models were systematically evaluated to ensure correspondence between model assumptions and each dataset. In addition, visual explorations of homogeneity of variance (homoscedasticity), model misspecification, and spatial correlation were conducted by plotting Pearson residuals against fitted values, explanatory variables and spatial correlation, respectively. The normality of the residuals and the model fit were verified through examination of a histogram of the Pearson residuals and a quantile-quantile (q-q) plot.

Since one of the main goals of this study is to examine the differences between circle hooks and traditional J-hooks, we considered the J (Mustad 9/0) as the reference level for comparisons between hook types and catch rates. The families used to fit the models were chosen based on the dispersion parameter, distribution of the response variable (number of individuals) for each species, zero frequency in the data, and visual inspection of diagnostic plots. The final model followed the backward model selection procedure, which typically starts with all candidate predictors and systematically removes variables based on their statistical significance or lack of contribution (S3 Table in S1 File). During the process, the performance of the models was also evaluated by considering the lowest value of Akaike Information Criterion (AIC). All analyses were performed using the mgcv package (1.6–1) [45] on the R statistical platform [46].

### Relative size selectivity

The same exploratory analyses described in the previous section (collinearity, outliers, zero inflation, and missing data) were performed before the models were run. The dataset for each species was modelled using GAMM with a binomial error distribution and logit link to verify the effects of explanatory variables, mainly hook type, on the distribution of individual body sizes of species caught.

The response variable (size) in each model was converted to a binomial category based on a size threshold for each species. The threshold dichotomizes the continuous probability output from the models into a binary result (0 or 1). The choice of the thresholds to binarize the response variable considered the approximated size of small juveniles for each species based on [47] for blue shark, [48] for swordfish, [49] for loggerhead sea turtle, and [50] for shortfin mako (Table 5). We also considered the balance among the distribution of observations for each hook type along the factors of the binary response variable, following the assumption that the data must be evenly distributed to perform a logistic regression. Individuals with sizes smaller than the cut-off value were classified as 0 and larger were categorized as 1. Therefore, the final models predicted the response probabilities of individuals smaller than the threshold size in relation to explanatory variables, mainly the hook type.

The models were also evaluated to ensure correspondence between model assumptions and each dataset (S6-S9 Figs in S1 File). For all the binomial GAMMs, the backward model selection was used considering mainly the significance and/or contribution of the variables to the model, especially the hook type effect (S4 Table in S1 File). The significance at the 0.05 confidence was measured with the chi-square test. Binomial GAMMs were performed using the mgcv package (1.6–1) [45] on the R statistical platform [46].

### Anatomical hook position

The anatomical hooking positions were grouped into three categories: external (entangled or foul-hooked on the body), mouth (lower or upper jaw), and deep-hooking (internally hooked in the throat, oesophagus, or deeper). This analysis is an important indicator of the degree of injury caused by each hook and consequently the probability of post-release survival [5, 23, 51].

Multinomial logistic regression models were carried out to evaluate effects of hook type on the anatomical hooking position over different body sizes. Multinomial regression is an extension of binary logistic regression that allows for more than two levels of dependent variable and is commonly used when the response variable is categorical [52]. Exploratory analyses were also conducted to ensure that the data are in accordance with the model’s assumptions, such as independence of observations, absence of multicollinearity, and independence of irrelevant alternatives (IAA–Hausman/McFadden test). One model was performed for each of the four species using "mouth" and "JM9" as the reference levels for comparisons among levels of hook location and hook type, respectively. All the analyses were performed using the R package nnet [53] on the R statistical platform.

## Results

### Effects of hook types on catch rates

The influence of explanatory variables on catch rates was different for the four species analysed in this study (GAMM) (Table 3).

**Table 3:** Final fitted models (GAMM) for the four species analysed in this study with the number of individuals as response variable.

Species	Model	Family	Link	ED (%)	DP
Blue shark	N.PGL ∼ intercept + *a* × Year + *b* × c × Hook type + *s*(SST) + *s*(SSHa) + *s*(Lon,Lat) + *s*(Date, bs = "re") + Offset(log(Effort))	Negative binominal	Log	95.90	1.07
Swordfish	N.XGL ∼ intercept + *a* × Year + *b* × Hook type + *s*(SST) + s(Lon) + s(Lat) + *s*(Date, bs = "re") + Offset(log(Effort))	Poisson	Log	50.08	0.97
Loggerhead sea turtle	N.CCA ∼ intercept + *a* × Hook type + *s*(SST) + *s*(Lon,Lat) + *s*(Date, bs = "re") + Offset(log(Effort))	Zero inflated Poisson (hurdle)	Identity	71.00	0.68
Shortfin mako	N.IOX ∼ intercept + *a* × Year + *b* × Hook type + *s*(SST) + *s*(LunarCycle) + *s*(Date, bs = "re") + Offset(log(Effort))	Zero inflated Poisson (hurdle)	Identity	61.20	0.69

ED = explained deviance, DP = dispersion parameter.

**Fig 2:**
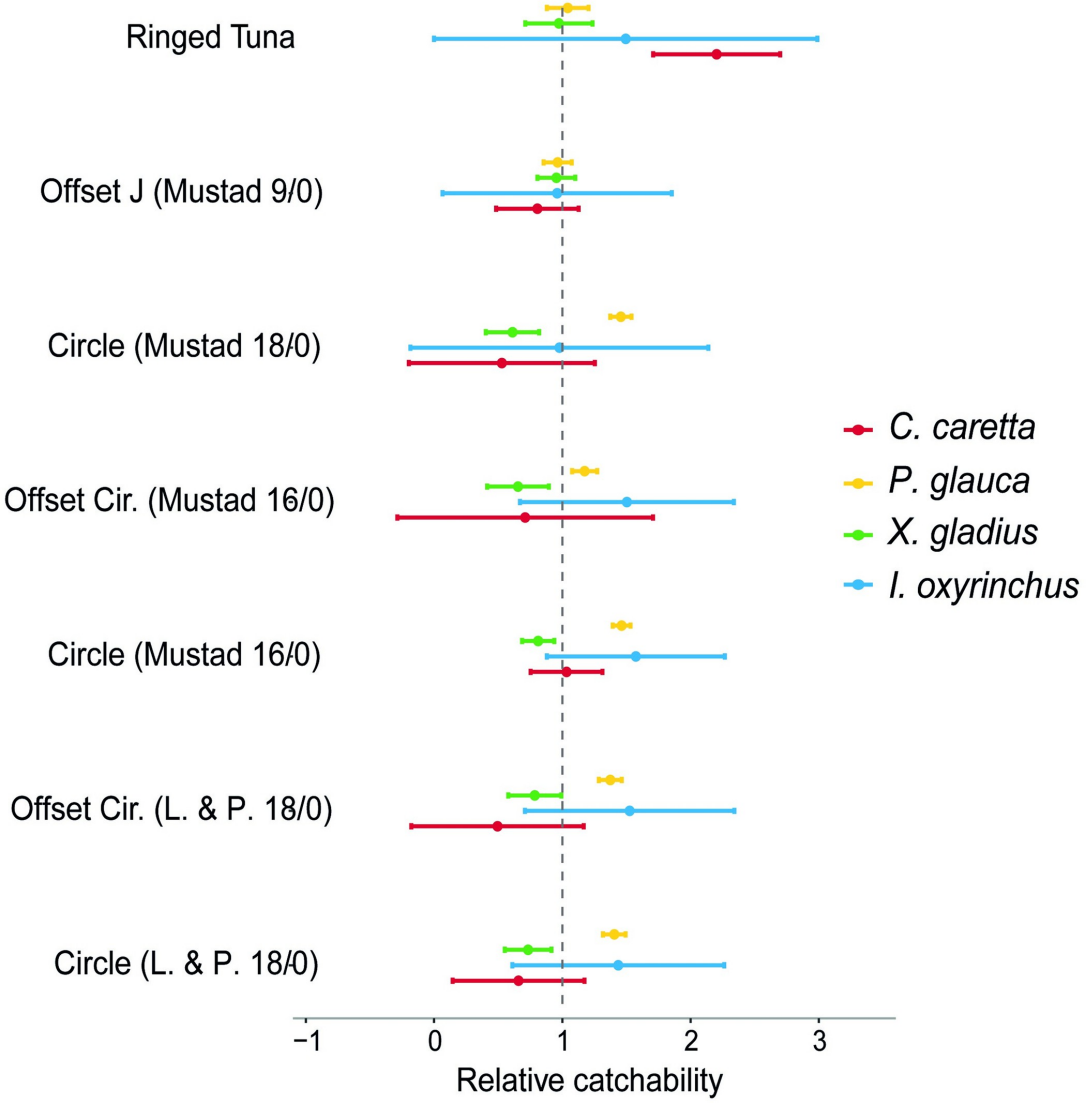
Relative catch rates for the four species using different hook types compared to J-hooks (Mustad 9/0). Solid dots represent the mean response calculated from the exponent of the GAM estimated hook type parameters. The horizontal lines are the 95% confidence intervals.

**Fig 3:**
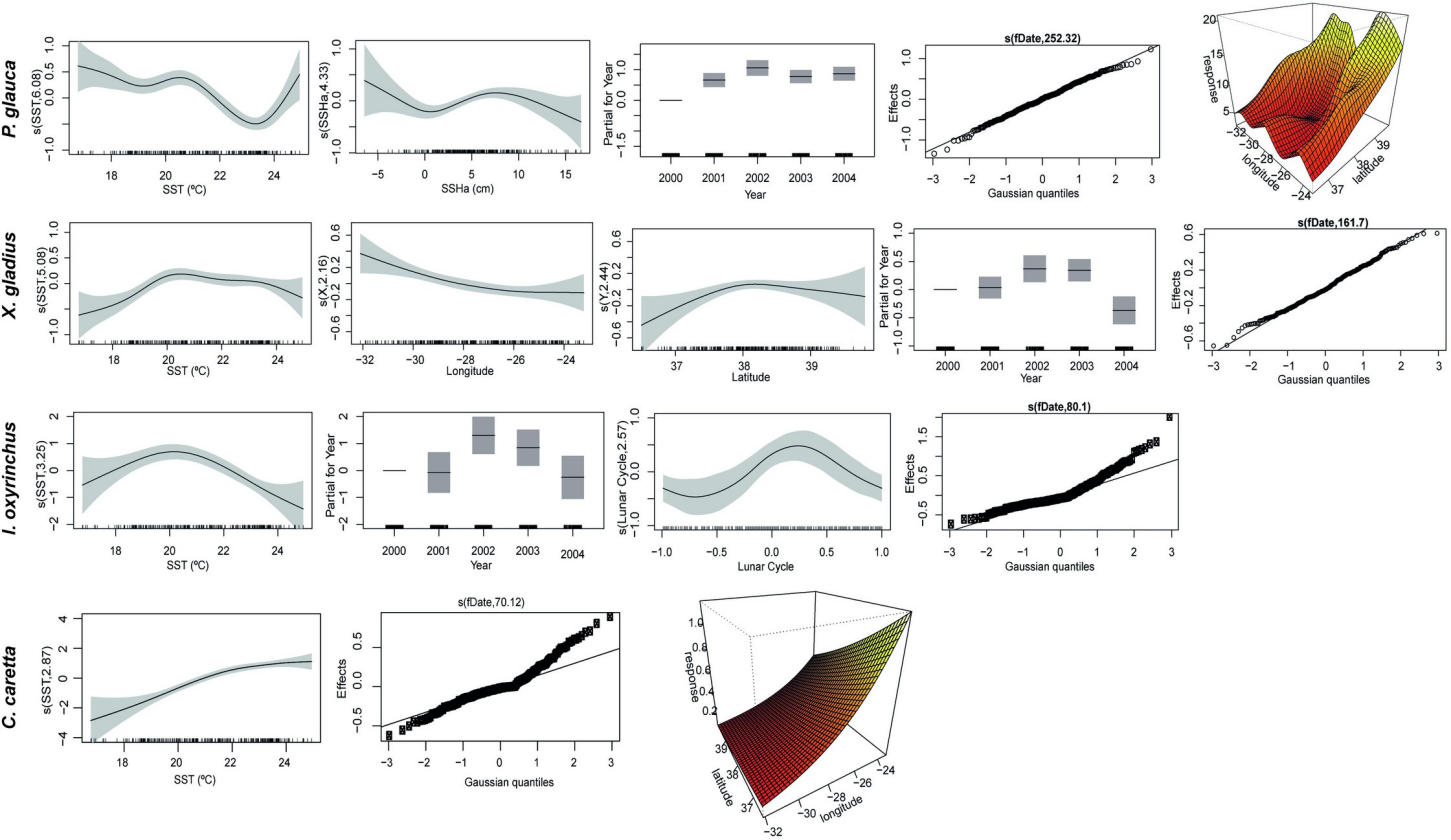
Additive fits of the explanatory variables in the final GAMMs for the four species analysed in this study. Thick marks on the x-axis of the independent variables indicate the distribution of observations. Shaded areas represent the 95% confidence intervals.

The catches of blue shark were higher for all types of circle hooks (offset and non-offset), mainly the straight CM18 and CM16 (Fig 2). The use of both hooks increases the chances of capturing this species by 46% (CI = 34–58%) when compared to the J-hook Mustad 9. The smooth terms in the model also provided a significant influence on the catch rates of blue sharks (Table 4). The peak occurred at SSTs between 20 and 22° C, with catch rates dropping at higher temperatures (Fig 3). The captures of blue shark were also influenced by SSHa, with a significant positive effect for SSHa values between 5 and 10 cm and a negative effect between −1 and 2 cm. The blue shark catch rates were higher at the end of autumn (November and December) and in the year 2002, when the wire leader was employed in the trial.

**Table 4:** Analysis of deviance (ANOVA) for the final GAMMs of the four species analysed in this study.

Species	Parametric terms				Approximate significance of smooth terms				
	Variable	df	Chi sq.	p-value	Variable	edf	Ref. Df	Chi sq.	p-value
Blue shark	Hook type	7	224.98	<0.001	s(SST)	6.08	6.21	99.42	<0.001
	Year	4	94.94	<0.001	s(SSHa)	4.44	4.45	18.21	0.002
					s(X,Y)	22.77	23.21	249.74	<0.001
					s(Date)	252.32	322.00	2262.40	<0.001
Swordfish	Hook type	7	39.33	<0.001	s(SST)	5.08	5.62	31.04	<0.001
	Year	4	62.18	<0.001	s(Date)	161.70	323.00	341.05	<0.001
					s(X)	2.16	2.44	17.01	<0.001
					S(Y)	2.44	2.79	7.25	0.035
Loggerhead sea turtle	Hook type	7	55.46	<0.001	s(SST)	2.87	3.42	82.87	<0.001
					s(X,Y)	2.00	2.00	75.32	<0.001
					s(Date)	70.12	327.00	121.56	<0.001
Shortfin mako	Hook type	7	2.66	0.915	s(SST)	3.18	3.77	29.09	<0.001
	Year	4	39.70	<0.001	s(Lunar Cycle)	2.70	8.00	57.45	<0.001
					s(Date)	72.42	324.00	132.57	<0.001

Four types of circle hooks showed a significant decrease in the catch rates of swordfish, a target species in the Azorean pelagic longline fishery (Fig 2). The lowest catch rates were observed for the CM18 hook, which caught 39% (CI = 25–50%) less than the JM9. No hook type produced a significant increase in captures relative to a J-hook (Mustad 9). The swordfish catch rates were also influenced by SST, reaching a peak around 22° C (Fig 3) (Table 4), and higher catch rates occurred in years 2002 and 2003.

Three types of circle hooks (CLM18, CLM18O and CLM16) showed significantly lower catch rates for loggerhead sea turtles compared to J (Mustad 9/0) hook (Fig 2). The CM18 hook was the gear that most avoided capturing loggerhead sea turtles as it caught around 58% (CI = 18–78%) fewer individuals compared to JM9. The catch rates using RT hook were around 136% (CI = 58–252%) higher compared to JM9, which makes it the most harmful hook for sea turtles. The catches tended to be higher at temperatures above 21° C, while the relationship between SSHa and *C*. *caretta* captures was slightly negative (Fig 3) (Table 4).

None of the hooks tested in this study showed differences in the shortfin mako catch rates compared to JM9. Circle hooks seemed to capture more individuals, but these relationships were not statistically significant (Fig 2) (Table 4). However, the catch rates of this species varied greatly in relation to the smooth terms used in the final model. Catch rates were influenced by SST, with a significant positive effect at temperatures around 20-21° C (Fig 3) (Table 4). The lunar cycle also influenced shortfin mako catches, which were higher shortly after the full moon. Similar to other species, the highest capture rates were recorded in 2002.

### Relative size selectivity

**Fig 4:**
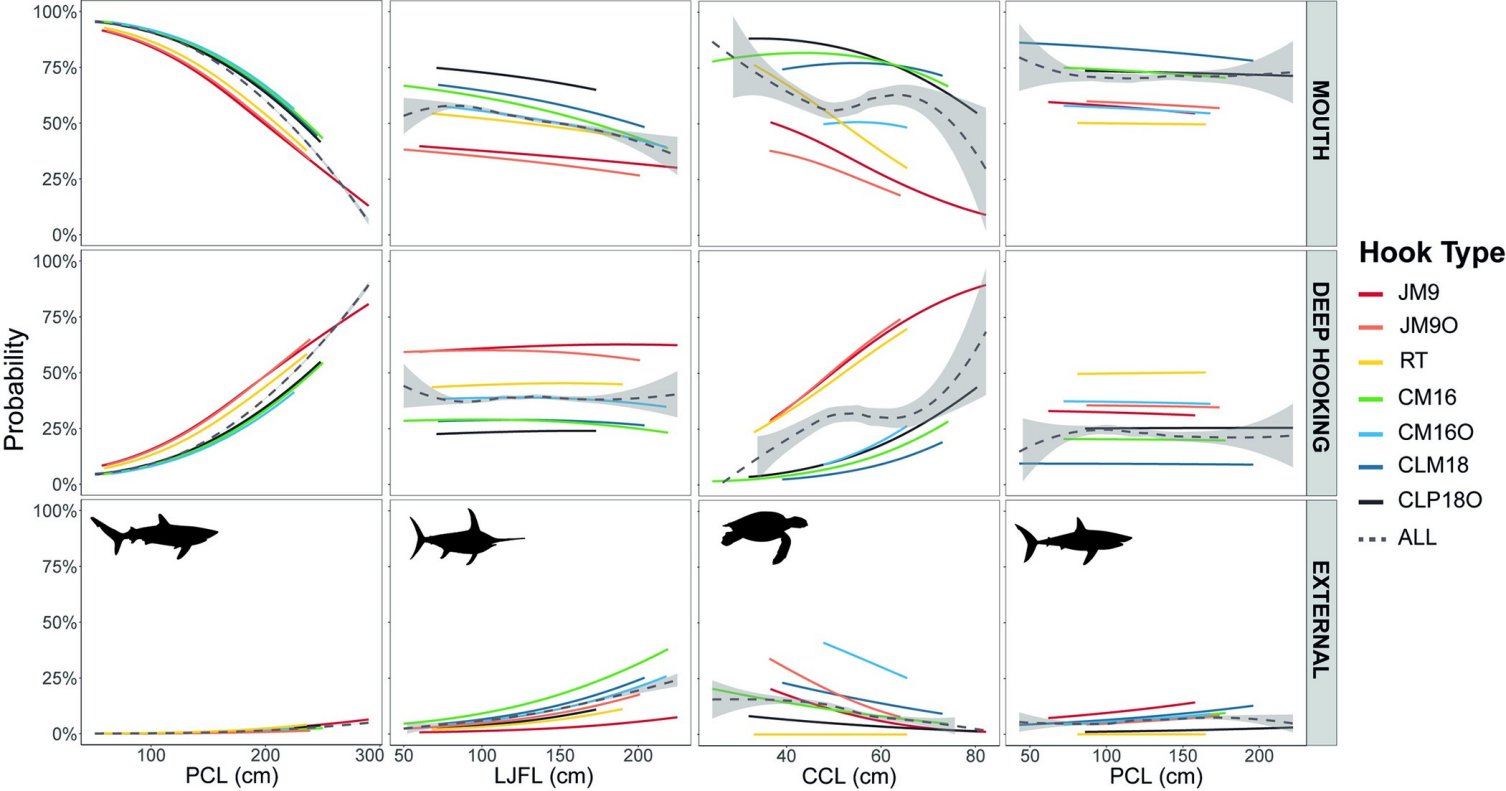
Predicted probabilities of the GAMMs binomial response for the four species caught by different hook types compared to J-hook (Mustad 9/0). The boxplots indicate the odds of each hook type catching individuals smaller than the threshold sizes established for each species (left panel). The right panel shows cumulative frequencies of the predicted response over species size. The dashed lines represent 50% of individuals retained below each threshold of species size. A: Blue shark. B: Swordfish. C: Loggerhead sea turtle. D: Shortfin mako. Asterisks indicate significant differences between hook types compared to J-hook (Mustad 9/0).

The final models (GAMMs) depicting the relationships between relative size selectivity and the explanatory variables are presented in Table 5. For the blue shark, circle hooks are more likely to capture individuals smaller than 125 cm when compared to other hooks (Table 6, Fig 4A and S6 Fig in S1 File). The JM9O was the most selective gear, retaining around 35% of individuals below 125 cm PCL.

**Table 5:** Final binomial fitted models (GAMMs) for the four species analysed in this study. The models predict the probability of individuals being smaller than the threshold size used in this study, based on values obtained from the literature. The link function used in the four models was “logit”. ED = explained deviance.

Species	Model	Threshold (cm)—Literature	ED (%)
Blue shark	PCL<125 ∼ intercept + *a* × Hook Type + *b* × Year + c × s(Lunar Cycle) + *s*(SST) + *s*(X,Y) + *s*(Date)	125	17.00
Swordfish	LJFL<115 ∼ intercept + *a* × Hook type + *s*(X) + s(Lunar Cycle) + *s*(Date)	111.7	5.00
Loggerhead sea turtle	CCL<55 ∼ intercept + *a* × Hook Type + *s*(X) + *s*(Date)	48–59	24.90
Shortfin mako	PCL<120 ∼ intercept + *a* × Hook type + *s*(Date)	100–150	21.40

**Table 6:** Analysis of deviance (ANOVA) for the final binomial GAMMs of the four species analysed in this study.

Species	Parametric terms				Approximate significance of smooth terms				
	Variable	df	Chi sq.	p-value	Variable	edf	Ref. Df	Chi sq.	p-value
Blue shark	Hook type	7	58.28	<0.001	s(SST)	1.70	1.78	44.31	<0.001
	Year	4	56.42	<0.001	s(Lunar Cycle)	4.02	8.00	1180.89	0.000
					s(X,Y)	14.95	15.95	101.37	<0.001
					s(Date)	208.96	313.00	838.06	<0.001
Swordfish	Hook type	7	14.17	0.048	s(Lunar Cycle)	2.23	8.00	18.41	0.000
					s(X)	1.00	1.00	4.60	0.032
					s(Date)	48.44	308.00	61.66	0.003
Loggerhead sea turtle	Hook type	7	24.46	0.001	s(X)	1.00	1.00	6.07	0.014
					s(Date)	37.34	150.00	53.16	0.002
Shortfin mako	Hook type	7	6.81	0.449	s(Date)	40.52	160.00	58.27	0.001

There is a tendency for the circle hooks to catch swordfish smaller than 115 cm LJFL. However, the only two types of hooks that showed significantly higher probabilities of capturing individuals below 115 cm were CLP18 and RT compared to JM9 gear (Table 6, Fig 4B and S7 Fig in S1 File). The odds of catching small loggerhead turtles were the opposite of the findings for blue shark (Fig 4C and S8 Fig in S1 File). For this sea turtle species, circle hooks (especially the CM16, CLP18, and CLP18O) showed lower probabilities of retaining individuals smaller than 55 cm CCL compared to JM9. No significant differences were found between the probabilities of capturing shortfin mako smaller than 120 cm for the different hook types, except for CLP18, which is less likely to retain individuals below this threshold size (Table 6, Fig 4D and S9 Fig in S1 File).

### Anatomical hook position and size

Multinomial logistic regression models were used to assess the effects of hook type on the anatomical hooking position over different sizes. The CLP18 and CM18, hooks with same width, were grouped into the CLM18 category to optimize the distribution of observations within the variable hook type. Effects of environmental variables were not analysed in these models.

**Fig 5:**
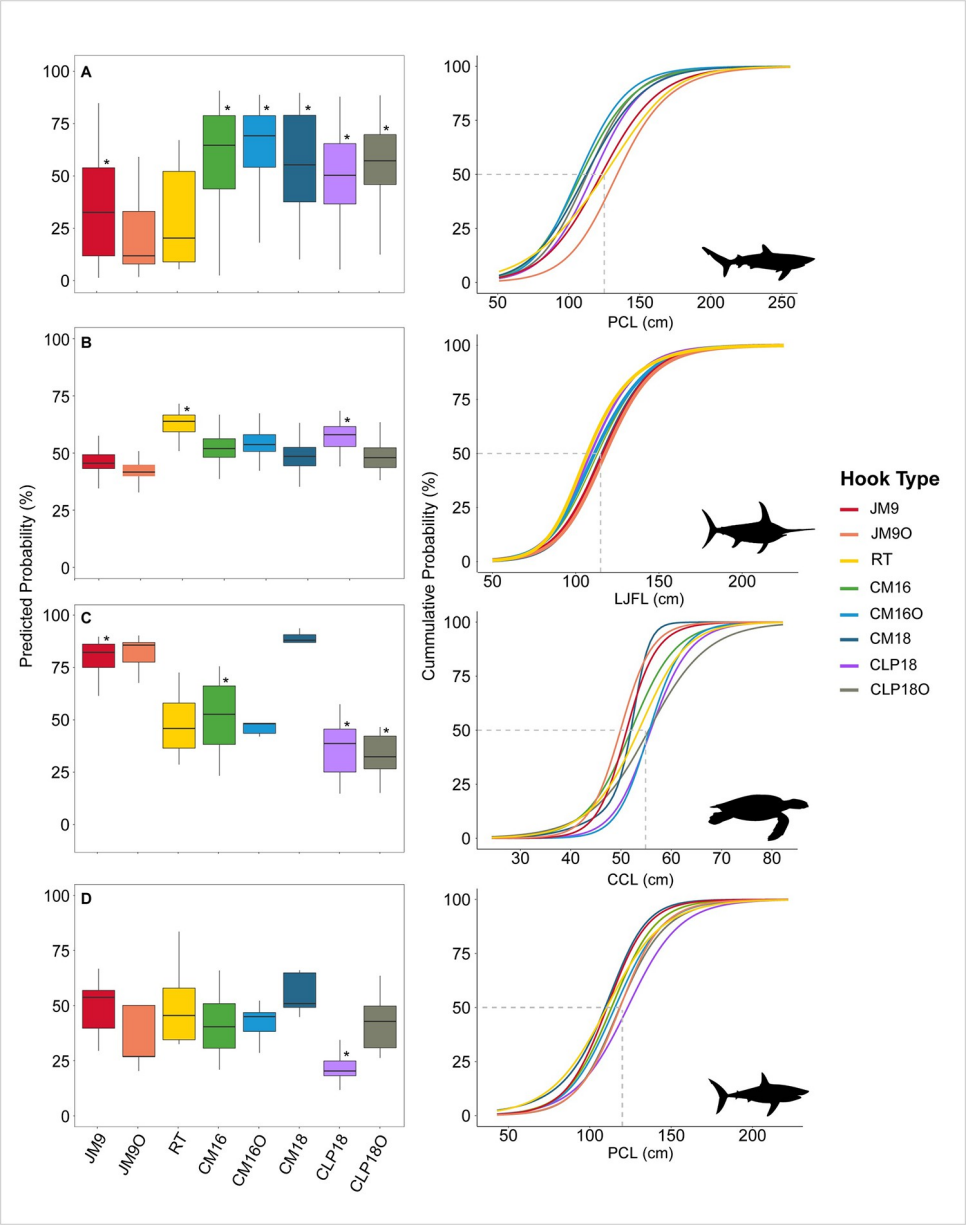
Distribution of the probabilities for all hook types to lodge in different parts of the animal body in relation to the size of the four species analysed. The overall effect of the hook is represented by the dashed line with the standard error shaded in grey.

In general, there is a tendency for the circle hooks to lodge in the mouth more than in deep-hooking positions, when compared to the JM9 hook (Fig 5). All circle hooks tested in this study showed a lower probability of catching blue shark and swordfish in deep positions in relation to the mouth. For blue shark, the odds ratio of catching animals deeply hooked using circle hooks was between 45–52% lower in comparison with animals hooked in the mouth, while for swordfish the decrease in these rates ranged from 57 to 98% using circle hooks (Table 7).

**Table 7:** The odds ratios for all hook types, compared to JM9, to lodge in different anatomical positions in relation to the mouth. The odds ratios (OR) were calculated from the exponent of the coefficients estimated in the multinomial logistic model.

	Blue shark			Loggerhead sea turtle			Swordfish			Shortfin mako		
Characteristic	OR	95% CI	p-value	OR	95% CI	p-value	OR	95% CI	p-value	OR	95% CI	p-value
**Deep Hooking**												
PCL/CCL/LJFL	1.02	1.02, 1.02	**<0.001**	1.06	1.03, 1.10	**<0.001**	1	1.00, 1.01	0.27	1	0.99, 1.01	0.95
Hook Type												
JM9	—	—		—	—		—	—		—	—	
CLM18	0.53	0.46, 0.61	**<0.001**	0.05	0.02, 0.14	**<0.001**	0.28	0.21, 0.35	**<0.001**	0.2	0.06, 0.62	**0.006**
CLP18O	0.55	0.47, 0.63	**<0.001**	0.09	0.03, 0.32	**<0.001**	0.2	0.14, 0.27	**<0.001**	0.61	0.23, 1.66	0.33
CM16	0.51	0.44, 0.58	**<0.001**	0.07	0.04, 0.15	**<0.001**	0.29	0.23, 0.37	**<0.001**	0.49	0.19, 1.24	0.13
CM16O	0.48	0.39, 0.58	**<0.001**	0.16	0.02, 1.65	0.12	0.43	0.30, 0.63	**<0.001**	1.16	0.42, 3.22	0.77
JM9O	0.97	0.77, 1.21	0.77	1.32	0.62, 2.80	0.48	1.06	0.78, 1.44	0.72	1.06	0.29, 3.92	0.92
RT	0.82	0.61, 1.10	0.18	0.67	0.33, 1.37	0.27	0.53	0.35, 0.79	**0.002**	1.77	0.29, 10.7	0.53
**External**												
PCL/CCL/LJFL	1.02	1.02, 1.03	**<0.001**	0.97	0.93, 1.02	0.28	1.02	1.01, 1.02	**<0.001**	1.01	0.99, 1.03	0.41
Hook Type												
JM9	—	—		—	—		—	—		—	—	
CLM18	0.44	0.21, 0.90	**0.024**	0.83	0.29, 2.32	0.72	2.87	1.43, 5.76	**0.003**	0.46	0.10, 2.11	0.32
CLP18O	0.66	0.32, 1.36	0.26	0.21	0.02, 1.77	0.15	1.51	0.67, 3.41	0.32	0.1	0.01, 1.04	**0.054**
CM16	0.41	0.20, 0.85	**0.017**	0.48	0.19, 1.19	0.11	4.21	2.16, 8.22	**<0.001**	0.44	0.11, 1.85	0.26
CM16O	0.6	0.22, 1.61	0.31	2.74	0.39, 19.4	0.31	2.89	1.19, 6.97	**0.019**	0.59	0.11, 3.31	0.55
JM9O	0.36	0.08, 1.60	0.28	2.22	0.77, 6.42	0.14	3.84	1.72, 8.60	**0.001**	0.49	0.04, 5.46	0.56
RT	1.03	0.29, 3.67	0.95	0	0.00, 0.00	**<0.001**	1.74	0.57, 5.30	0.33	0	0.00, 0.00	**<0.001**

With the exception of the CM16O hook, all circle hooks also showed lower odds ratios to be deep-hooked, compared to the mouth, in loggerhead sea turtle. The same tendency was also verified for the shortfin mako, although the only significant relationship was for the CLM18 hook (Table 7).

The probability of the hook being swallowed (deep-hooking) increased with the size of the blue shark (z = 27.38, p < 0.001), especially for the J-type hooks (Fig 5). The use of circle hooks reduces the probability of ingestion mostly for larger animals compared to J and RT hooks, while smaller individuals are mostly hooked in the mouth with all gear types. For swordfish, the larger animals tend to be hooked externally (z = 5.84, p < 0.001). The odds of the swordfish being deep-hooked remains relatively constant over the animal size. However, RT and J-hooks have more than 50% chance of being swallowed. Larger turtles were more likely to be hooked internally (z = 3.37, p < 0.001), mainly when JM9, JM9O, and RT hooks were used. The introduction of circle hooks reduces the odds of ingestion almost to zero for sea turtles below 45 cm. The gain is highest for large turtles (around 80 cm) where the probability of ingestion is reduced by almost 50% when using circle hooks. The relationship between deep-hooking and size did not vary significantly with size for shortfin mako. However, for this species, the J and RT hooks were more likely to lodge internally compared to the circle ones. Especially when the CLM18 hook type is used, the chances of being deep-hooked is near to zero.

## Discussion

The multi-year fishery trial within the Azorean EEZ was one of the most comprehensive studies on the effects of gear modification on target and bycatch megafauna in pelagic longline fisheries. The study tested eight types of circle, ringed tuna, and J hooks, including offset and non-offset, which caught 27,603 individuals of 34 species over five years. The four most abundant species (*P*. *glauca*, *C*. *caretta*, *X*. *gladius*, and *I*. *oxyrinchus*) accounted for 96.5% of the total catches and were analysed for the influence of the hook types on catch rates, relative size selectivity and anatomical hooking position. In general, the circle hooks were more efficient in avoiding captures of the loggerhead sea turtle and swordfish, while they showed higher catch rates for the blue sharks. Although circle hooks demonstrated a tendency to catch more shortfin mako sharks compared to conventional hooks, this increase was not statistically significant. The circle hooks also showed high probabilities of catching juvenile blue sharks and were related to low catches of small loggerhead turtles. Moreover, for the four species, circle hooks were more likely to lodge in the mouth than in deeper anatomical positions (throat, oesophagus, stomach), when compared to J (Mustad 9/0) hooks. These results highlight the role of circle hooks in reducing hooking-related injury and post-release mortality [4, 20, 23, 30, 54–56].

The blue shark has become one of the main target species of the pelagic longline fishery in the Azores, especially during the spring when swordfish catches are low [7, 57]. This species dominated the catch composition in this study, which reflects the high abundance already documented for blue sharks in Azorean waters [58, 59]. It is considered a resilient species due to its relative fast growth and large number of offspring [60]. In addition, the Azores region is an important nursery area for this species, although there is spatial and seasonal segregation in the population distribution [38]. The combination of these factors could explain the vulnerability of blue sharks to be captured by pelagic longline fisheries in the Azores.

Although there is some discrepancy among studies, in general, shark species have higher risk of being caught with circle hooks than J-shaped hooks, especially *P*. *glauca*, the dominant elasmobranch species caught in many open ocean pelagic longline fisheries [61–64]. In the present study, blue shark catches increased between 17% and 46% (mean 37%) when using circle hooks compared to JM9. These results agree with a meta-analysis conducted by [20], which found that circle hooks have 46% higher catch rates for six shark species, including blue sharks, relative to traditional J-hooks. On the other hand, [65] verified a reduction in blue shark captures with circle hooks in the Hawaii-based tuna longline fishery and [28, 56] found no statistical differences between the hook types (circle and traditional) used to catch blue shark in a Japanese and South Adriatic Sea longline trials, respectively.

None of the hooks tested in this study showed significant differences in the *I*. *oxyrinchus* catch rates compared to J (Mustad/9). Several studies have shown mixed effects when considering the use of different hook types on shortfin mako catch rates. In general, the circle hooks tend to increase the *I*. *oxyrinchus* catches [25, 66]; however, in most cases, this increase in catches is not significant [32, 67, 68]. These different results found in the sharks studies might be associated with factors conditioned to each fishing/trial, such as bait type, leader type, setting time, as well as the intrinsic population behaviours influenced by local environmental conditions (thermoregulatory vertical migrations, foraging habits, predator avoidance, etc.) [69, 70].

A significant decrease in the catch rates using circle hooks was verified for swordfish and sea turtles, especially the CM18 type, which caught around 39% and 58% fewer swordfish and loggerhead turtles, respectively, when compared to J (Mustad 9/0). Several longline fishery trials evaluating the effect of gear modification on catch rates found similar results for these two species [32, 56, 71, 72]. In the Equatorial Atlantic, [73] found a reduction in the swordfish catch rates of approximately 25% when changing from J to circle hooks. In the Western North Atlantic pelagic longline fishery, [4] found that the combination of 18/0 circle hooks baited with mackerel reduced loggerhead catch by 88% compared to traditional J-hooks using squid as bait. The same study reported swordfish catch rates increased by 17% on 18/0 circle hooks with mackerel bait and decreased 31% with the same hook baited with squid. The effectiveness of circle hooks combined with mackerel bait was also verified in many other studies conducted in pelagic longline fisheries in different zones of the North and South Atlantic Ocean [68, 74], Hawaii [24], and Western North Pacific [75]. Therefore, it seems that the combination of 18/0 circle hook with fish bait is appropriate for the pelagic longline fisheries targeting swordfish and blue shark, since it reduces bycatch of loggerhead sea turtles and probably increases or maintains the catch rates of the target species [74]. In the present study, we did not evaluate the effects of different types of bait on the captures, so more trials need to be carried out to confirm whether this combination of factors is indeed effective in reducing bycatch in the Azores fisheries.

It is important to highlight that the RT hook was the most harmful to loggerhead sea turtles, resulting in significantly higher numbers of captures compared to other hook types. Furthermore, it is likely that this type of gear could deeply hook more than 50% of individuals over 50 cm. Although this result is consistent with other studies [23, 65, 71], we should exercise caution in its interpretation since, in this trial, the RT hook was not directly compared with all other hooks in the same year. The high catch rates of RT hook were so detrimental to the sea turtles that its use was discontinued after one month of the trial. Therefore, the use of this gear in Azorean pelagic longline fisheries is highly discouraged.

The year effect had a significant impact on catch rates for all species, except for loggerhead sea turtles, which is coincident with the leader type used each year in the trials. The use of wire leaders has been found to increase catch rates and reduce haulback survival for most elasmobranch species caught in pelagic longline fisheries [5, 76, 77]. Most of the shark species have sharp teeth, enabling them to sever monofilament leaders and escape [61]. In order to reduce shark mortality, the wire leader has been banned in some pelagic longline fisheries [78], including in the Azores within 100 nm off the islands. While most of studies indicate that catch rates for swordfish are higher when using monofilament leaders [7, 68, 77], our study revealed slightly higher catch rates when employing wire leaders, with the exception of 2004. These findings are likely attributed to the varying combinations of hook types and wire leaders used each year, particularly in 2004 when only wider circle hooks (CLP18 and CLP18O) were employed.

The effects of hook types on the relative size selectivity have been divergent, especially for sharks, with some studies showing significant results on mean length at capture, while others found no significant differences between the species size and hook types [25]. In this trial, circle hooks showed high probability of catching small blue sharks compared to JM9, the same result found in the Gulf of Mexico longline fishery [79]. However, in the western Atlantic, [80] reported that specimens caught by circle hooks are larger than those caught by the J-style, while no significant differences were found in other pelagic longline fisheries [28, 30].

Although this study showed a tendency for the CLP18 and RT to catch more small swordfish, in general, other studies have presented no effect of hook types on length at capture for this species [3, 30, 80]. Regarding sea turtles, almost all circle hook types are related to low catches of small individuals, mainly the larger CLP18 and CLP18O hooks. This result is in agreement with size-selectivity studies performed for this species [23, 32, 31], which affirm that larger gears reduce turtle capture and prevent smaller individuals, with relatively small mouths, from ingesting the hook. Except for CLP18O hook, no effect of hook type on relative size selectivity of shortfin mako was verified in the Azorean pelagic longline fishery, which corroborates the main findings from other studies [25, 30].

The use of circle hooks is related to a significant change in the anatomical hooking location for the majority of species caught in the pelagic longline fisheries [20, 23, 25]. The anatomical hooking position directly influences the survival at haulback since the jaw-hooking, compared to deep-hooking, reduces the animal injury and allows it to continue to swim and breathe while on the line [3]. The pelagic longline trial in the Azores showed that there is a tendency for the circle hooks to lodge in the mouth more than in deep-hooking positions when compared to the traditional J-hook. The four circle hooks tested in this study were hooked in the mouth more than in the throat, oesophagus or stomach for blue sharks and swordfishes and three of them were more likely to lodge in the loggerhead turtle mouth. These results are consistent with several studies on the effects of gear modification on post release survival of bycatch in longline fisheries [3, 32, 54, 67, 74].

A compilation of studies [23] concluded that circle hooks result in a lower proportion of hard-shelled and leatherback turtles swallowing the hook deeply (oesophagus and deeper), and therefore increase survival rates when compared to J-shaped hooks. Furthermore, a review of by-catch mitigation measures carried out by [61] based on 30 studies showed that although circle hooks increase elasmobranch catches, they reduce post-hooking mortality and deep hooking relative to J-hooks, which might benefit sharks caught in regulated fisheries (e.g. fishing quota or minimum size limits) by increasing the number of sharks released alive. In addition, the probability of deep hooking increases more rapidly with blue shark size for J-hooks compared to circle hooks. This highlights the importance of implementing circle hooks for the protection of juvenile animals, which is relevant because of the nursery role of the Azores for this species [38].

Sea surface temperature (SST) was the environmental factor that explained part of the variability in catch rates for all species analysed in this study. The peak of blue shark catch occurred in periods of lower temperatures (beginning of winter in December) at SSTs between 20 and 22° C. The catch rates tend to drop at higher temperatures, which is equivalent to the summer months (August and September). A similar trend in the relationship between catch rates and SST was verified for the shortfin mako. The fluctuations in blue shark abundance are mainly due to the changes in the spatial and temporal distribution of populations in terms of size and sex derived from the complex migration patterns of this species [38, 57].

Swordfish captures increased with SST and remained relatively constant at temperatures above 22°C. This fishery seasonality is contrary to that recorded for the blue shark. These differences in swordfish catch rates may be related to the distinct migratory dynamics and seasonality of fishing efforts in the Azores region, which is lower from September to October and higher between May and July [7]. It is worth highlighting that this trial was conducted from July to December and the high catch rates for blue shark have been reported mostly during spring in months not sampled in this study [7, 38, 58].

The loggerhead sea turtle catches tend to increase with higher SST [81–83]. Although the suitable SST range for loggerheads is between 18 and 21° [84, 85], the sea turtles in the Azores are possibly more attracted to the fishing gear due to scarcity of food in oligotrophic waters at temperatures above 21°C, which may explain higher catches in warmer temperatures.

## Conclusion

The present study demonstrated that the use of circle hooks could mitigate the impact of pelagic longline fisheries by decreasing the bycatch of sea turtles and reducing animal injuries caused by deep hooking. In addition, the use of these gears reduces injuries caused by deep-hooking, which allows for a higher post-release survival rate.

In the Azorean EEZ, the local government already recommended the use of circle hooks with a 30 mm minimum gap for pelagic longline fishery, although this gear replacement has been gradual since 2020. Additionally, the local government has recently implemented the prohibition of wire leaders within the 100 NM limit off the Azores. Although the use of circle hooks increases blue shark catches, the ban of wire leaders might be beneficial for these animals, since several studies have demonstrated that nylon leaders could be more easily severed by sharks and increase or maintain swordfish catches.

The use of circle hooks should be seen as a piece of the puzzle and complementary measures must be taken into account in order to protect bycatch and target megafauna, especially sharks. The sustainable integrated approach must also consider measures such as minimum legal catch size and the use of fish instead of squid as bait, which could minimize the bycatch of sea turtles and optimize the catches of target species. The combination of these recommendations with other strategies (reduce soak time, avoid spatio-temporally predictable bycatch hotspots, and establish quotas for target species) should also be tested to ensure the sustainable exploitation of fisheries resources in the region. Together, these management measures can significantly improve the sustainability of pelagic longline fishery in the Azores, which brings ecological, sociocultural, and economic benefits.

## Supporting information

S1 File Supporting tables and figures. S1 Table, The total number of hooks per year used in the GAMM analysis to examine the impact of different hook types on the catch rates of the four species. S2 Table, List of species captured during the trials conducted from 2000 to 2004, including the percentage of each species in relation to the total catches and their respective catch per unit effort (CPUE–ind/1000 hooks). S3 Table, Summary steps of the backward model selection process, including the initial full model and final model for each species. The final models are highlighted in bold. ED = explained deviance, NS = non-significant variables. S4 Table, Summary steps of the backward model selection process, including the initial full model and final model for each species. The final models are highlighted in bold. ED = explained deviance, NS = non-significant variables. S1 Fig, Correlation analysis with all candidate variables to perform the generalized additive mixed model (GAMMs) analyses. S2-S5 Figs, Diagnostic plots of the final GAMM model for blue shark, swordfish, loggerhead, and shortfin mako, respectively. Top left panel: normal QQ-plot of deviance residuals against theoretical quantiles; top right panel: histogram of the Pearson residuals; bottom left: residuals against the linear predictor; and bottom right: plot of observed vs fitted values. S6-S9 Figs, A—Correlation analysis with all candidate variables to perform the generalized additive mixed model (GAMMs) analyses (blue shark, swordfish, loggerhead sea turtle, and shortfin mako, respectively). B—Diagnostic plots of the final GAMM binomial model. Top left panel: normal QQ-plot of deviance residuals against theoretical quantiles; top right panel: histogram of the Pearson residuals; bottom left: residuals against the linear predictor; and bottom right: plot of observed vs fitted values.
(DOCX)
