# Correction: Effects of gear modifications in a North Atlantic pelagic longline fishery: A multiyear study

**DOI:** 10.1371/journal.pone.0315684

**Published:** 2024-12-09

**Authors:** Françoise D. Lima, Hugo Parra, Rita B. Alves, Marco A. R. Santos, Karen A. Bjorndal, Alan B. Bolten, Frederic Vandeperre

The images for Figs 4 and 5 are incorrectly switched. The image that appears as Fig 4 should be Fig 5, and the image that appears as Fig 5 should be Fig 4. The figure captions appear in the correct order. Please see the correct Figs 4 and 5 here.

**Fig 4:**
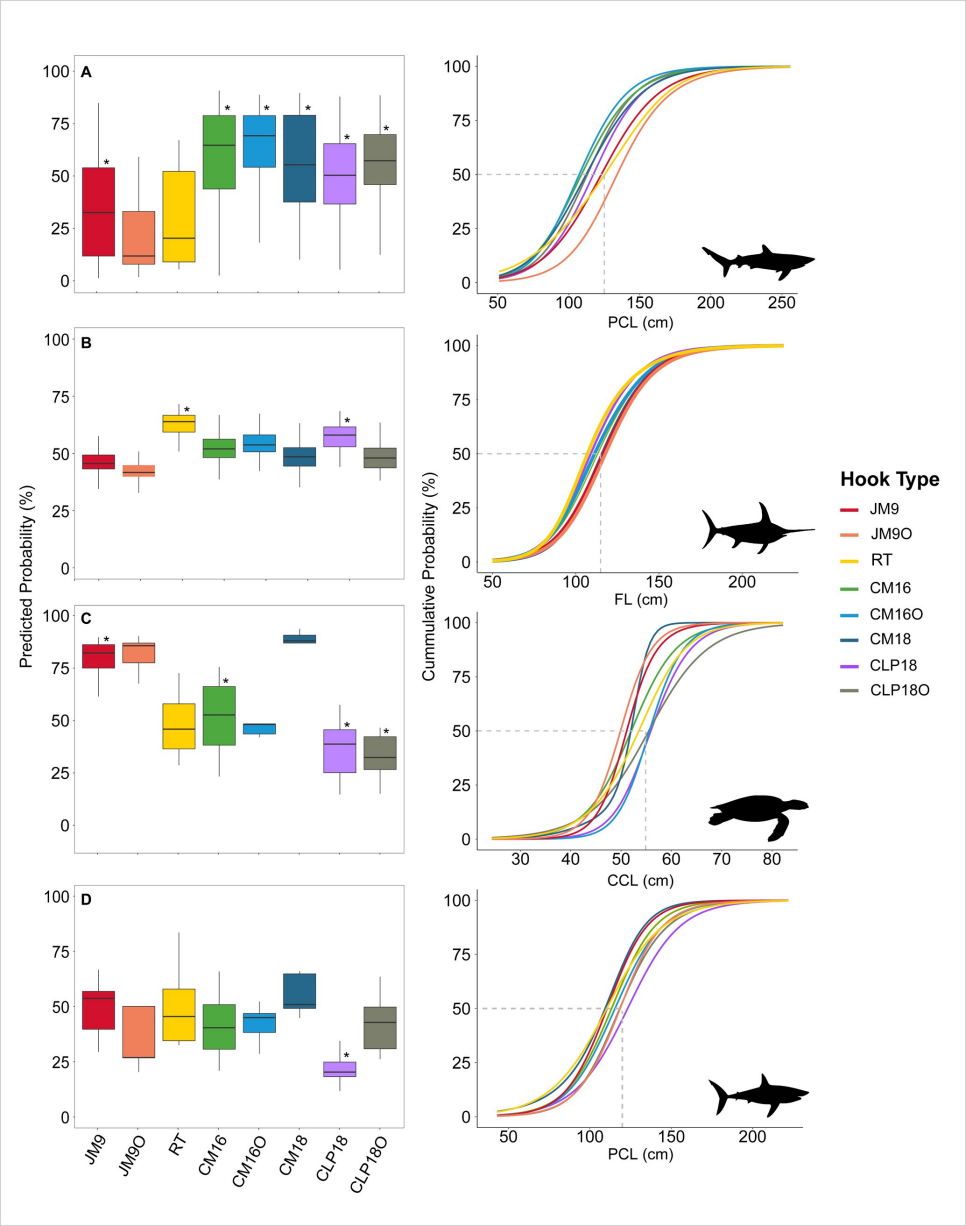
Predicted probabilities of the GAMMs binomial response for the four species caught by different hook types compared to J-hook (Mustad 9/0). The boxplots indicate the odds of each hook type catching individuals smaller than the threshold sizes established for each species (left panel). The right panel shows cumulative frequencies of the predicted response over species size. The dashed lines represent 50% of individuals retained below each threshold of species size. A: Blue shark. B: Swordfish. C: Loggerhead sea turtle. D: Shortfin mako. Asterisks indicate significant differences between hook types compared to J-hook (Mustad 9/0).

**Fig 5:**
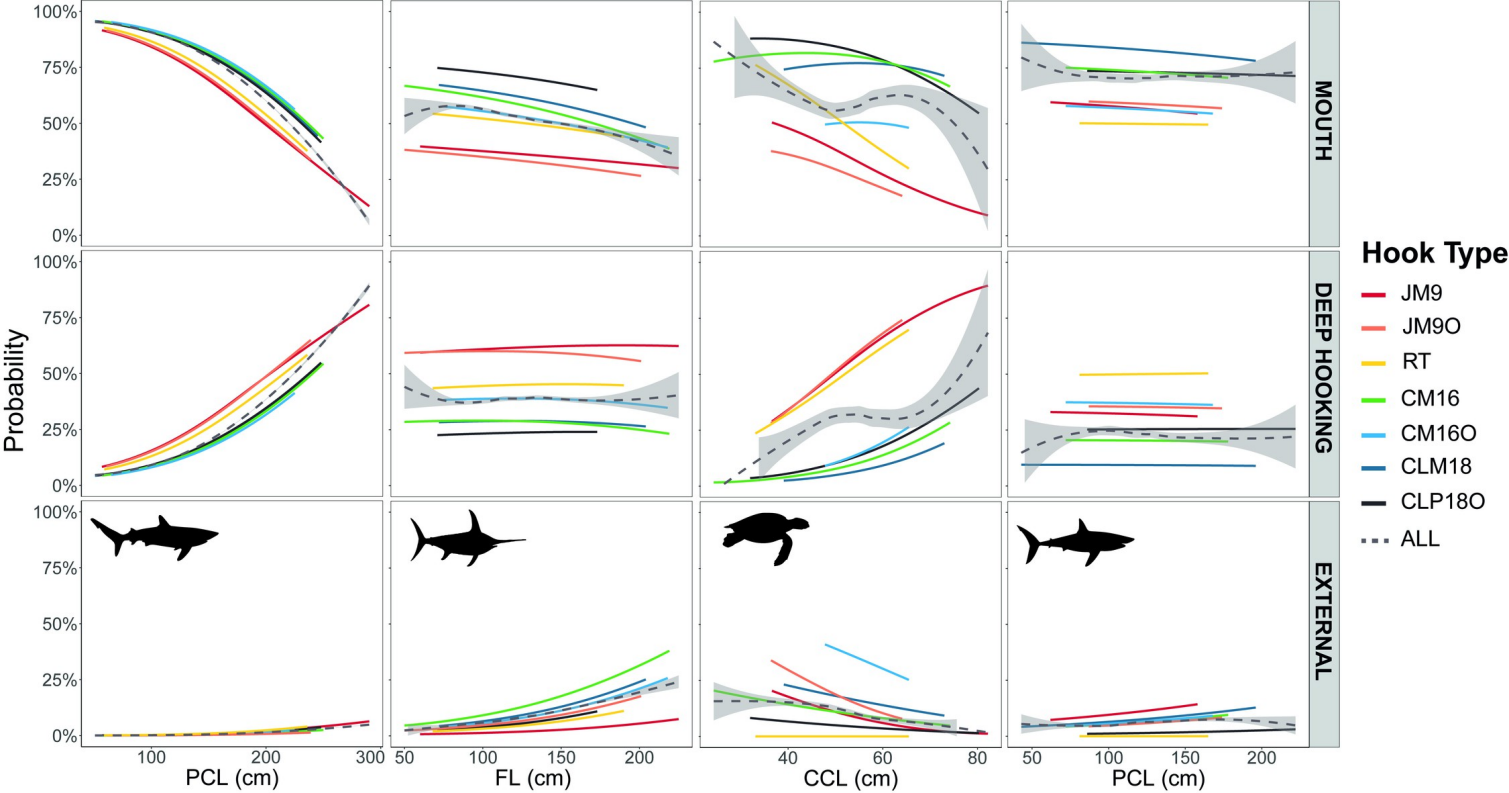
Distribution of the probabilities for all hook types to lodge in different parts of the animal body in relation to the size of the four species analysed. The overall effect of the hook is represented by the dashed line with the standard error shaded in grey.
