# Supplementary material for: Effects of gear modifications in a North Atlantic pelagic longline fishery: A multiyear study
Source: PLoS One. 2023 Oct 19;18(10):e0292727. doi: 10.1371/journal.pone.0292727 (PMC10586631; doi:10.1371/journal.pone.0292727)
Supplement: S1 File — S1 Table, The total number of hooks per year used in the GAMM analysis to examine the impact of different hook types on the catch rates of the four species. S2 Table, List of species captured during the trials conducted from 2000 to 2004, including the percentage of each species in relation to the total catches and their respective catch per unit effort (CPUE–ind/1000 hooks). S3 Table, Summary steps of the backward model selection process, including the initial full model and final model for each species. The final models are highlighted in bold. ED = explained deviance, NS = non-significant variables. S4 Table, Summary steps of the backward model selection process, including the initial full model and final model for each species. The final models are highlighted in bold. ED = explained deviance, NS = non-significant variables. S1 Fig, Correlation analysis with all candidate variables to perform the generalized additive mixed model (GAMMs) analyses. S2-S5 Figs, Diagnostic plots of the final GAMM model for blue shark, swordfish, loggerhead, and shortfin mako, respectively. Top left panel: normal QQ-plot of deviance residuals against theoretical quantiles; top right panel: histogram of the Pearson residuals; bottom left: residuals against the linear predictor; and bottom right: plot of observed vs fitted values. S6-S9 Figs, A—Correlation analysis with all candidate variables to perform the generalized additive mixed model (GAMMs) analyses (blue shark, swordfish, loggerhead sea turtle, and shortfin mako, respectively). B—Diagnostic plots of the final GAMM binomial model. Top left panel: normal QQ-plot of deviance residuals against theoretical quantiles; top right panel: histogram of the Pearson residuals; bottom left: residuals against the linear predictor; and bottom right: plot of observed vs fitted values. (DOCX) [file pone.0292727.s001.docx]

**SUPPORTING INFORMATION**

**Effects of gear modifications in a North Atlantic pelagic longline fishery: a multiyear study**

**Françoise D. Lima ^1,2*^, Hugo Parra ^1,2^, Rita B. Alves ^3^, Marco A. R. Santos ^4^, Karen A. Bjorndal ^5^, Alan B. Bolten ^5†^, Frederic Vandeperre ^1,2^**

^1^ IMAR – Institute of Marine Research, Departamento de Oceanografia e Pescas, Universidade dos Açores, Horta, Portugal

^2^ Institute of Marine Sciences - Okeanos, – Departamento de Oceanografia e Pescas, Universidade dos Açores, Horta, Portugal.

^3^ ISPA - Instituto Universitário de Ciências Psicológicas, Sociais e da Vida, Lisboa, Portugal

**^4^** DROTRH – Direção Regional do Ordenamento do Território e dos Recursos Hídricos, Governo dos Açores, Horta, Portugal.

^5^ ACCSTR – Archie Carr Center for Sea Turtle Research and Department of Biology, University of Florida, Gainesville, FL, USA

**^†^** *In memoriam*

S1 Table. The total number of hooks per year used in the GAMM analysis to examine the impact of different hook types on the catch rates of the four species.

| **Hook Type** | **Year** | | | | | **Total** |
| --- | --- | --- | --- | --- | --- | --- |
|  | **2000** | **2001** | **2002** | **2003** | **2004** |  |
| CLP18 |  |  |  | 50917 | 36096 | 87013 |
| CLP18O |  |  | 25153 |  | 36096 | 61249 |
| CM16 | 45075 | 28419 | 25153 | 50393 |  | 149040 |
| CM16O |  |  | 25153 |  |  | 25153 |
| CM18 |  | 28419 |  |  |  | 28419 |
| JM9 | 45075 | 28419 |  |  |  | 73494 |
| JM9O | 45075 |  |  |  |  | 45075 |
| RT |  |  |  | 12762 |  | 12762 |

S2 Table. List of species captured during the trials conducted from 2000 to 2004, including the percentage of each species in relation to the total catches and their respective catch per unit effort (CPUE – ind/1000 hooks).

| **Species** | **Year** | | | | | **Total** | **Percentage** | **CPUE** |
| --- | --- | --- | --- | --- | --- | --- | --- | --- |
|  | **2000** | **2001** | **2002** | **2003** | **2004** |  |  |  |
| *Prionace glauca* | 2089 | 3950 | 6599 | 5190 | 4710 | 22538 | 81.651 | 45.268 |
| *Xiphias gladius* | 953 | 537 | 549 | 875 | 311 | 3225 | 11.684 | 6.477 |
| *Caretta caretta* | 232 | 44 | 18 | 143 | 54 | 491 | 1.779 | 0.986 |
| *Isurus oxyrinchus* | 59 | 32 | 142 | 115 | 38 | 386 | 1.398 | 0.775 |
| *Alepisaurus spp.* | 26 | 49 | 20 | 37 | 60 | 192 | 0.696 | 0.386 |
| *Thunnus alalunga* | 8 | 12 | 18 | 83 | 25 | 146 | 0.529 | 0.293 |
| *Coryphaena hippurus* | 22 | 47 | 20 | 36 | 13 | 138 | 0.500 | 0.277 |
| *Alopias superciliosus* | 18 | 19 | 11 | 58 | 21 | 127 | 0.460 | 0.255 |
| *Thunnus obesus* | 17 | 7 | 23 | 60 | 8 | 115 | 0.417 | 0.231 |
| *Ruvettus pretiosus* | 17 | 23 | 1 | 7 | 5 | 53 | 0.192 | 0.106 |
| *Masturus lanceolatus* | 10 | 4 | 1 | 4 | 8 | 27 | 0.098 | 0.054 |
| *Mola mola* | 16 | 0 | 0 | 5 | 1 | 22 | 0.080 | 0.044 |
| *Lepidocybium flavobrunneum* | 5 | 8 | 3 | 3 | 2 | 21 | 0.076 | 0.042 |
| *Tetrapturus pfluegeri* | 10 | 1 | 1 | 3 | 2 | 17 | 0.062 | 0.034 |
| *Taractes* cf. *rubescens* | 6 | 4 | 1 | 2 | 2 | 15 | 0.054 | 0.030 |
| *Dasyatis violacea* | 4 | 5 | 0 | 4 | 2 | 15 | 0.054 | 0.030 |
| *Dermochelys coriacea* | 4 | 1 | 3 | 5 | 2 | 15 | 0.054 | 0.030 |
| *Galeorinus galeus* | 13 | 0 | 0 | 0 | 0 | 13 | 0.047 | 0.026 |
| *Katsuwonus pelamis* | 2 | 1 | 4 | 1 | 2 | 10 | 0.036 | 0.020 |
| *Lagocephalus lagocephalus* | 0 | 5 | 0 | 1 | 0 | 6 | 0.022 | 0.012 |
| *Sphyrna zygaena* | 1 | 0 | 1 | 4 | 0 | 6 | 0.022 | 0.012 |
| *Diodon hystrix* | 0 | 1 | 1 | 3 | 0 | 5 | 0.018 | 0.010 |
| *Heptranchias perlo* | 1 | 1 | 1 | 2 | 0 | 5 | 0.018 | 0.010 |
| *Thunnus albacares* | 2 | 1 | 0 | 0 | 1 | 4 | 0.014 | 0.008 |
| *Isurus paucus* | 0 | 0 | 0 | 0 | 2 | 2 | 0.007 | 0.004 |
| *Conger conger* | 1 | 0 | 0 | 0 | 0 | 1 | 0.004 | 0.002 |
| *Pagellus bogaraveo* | 1 | 0 | 0 | 0 | 0 | 1 | 0.004 | 0.002 |
| *Scomber japonicus* | 0 | 0 | 0 | 1 | 0 | 1 | 0.004 | 0.002 |
| *Polyprion americanus* | 0 | 0 | 1 | 0 | 0 | 1 | 0.004 | 0.002 |
| *Carcharhinus galapagensis* | 1 | 0 | 0 | 0 | 0 | 1 | 0.004 | 0.002 |
| *Manta birostris* | 0 | 0 | 0 | 0 | 1 | 1 | 0.004 | 0.002 |
| *Mobula tarapacana* | 0 | 0 | 0 | 1 | 0 | 1 | 0.004 | 0.002 |
| *Chelonia mydas* | 1 | 0 | 0 | 0 | 0 | 1 | 0.004 | 0.002 |
| *Larus cachinnans atlantis* | 0 | 1 | 0 | 0 | 0 | 1 | 0.004 | 0.002 |

**EFFECTS OF HOOK TYPES ON CATCH RATES - BACKWARD MODEL SELECTION**

**Abbreviations:**

N.IND: number of individuals

Y: year

M: month

HT: hook type

SST: sea surface temperature

SSHa: sea surface height anomalies

LC: lunar cycle

X1: longitude

Y1: latitude

NHOOK: number of hooks

NLL: Leader type


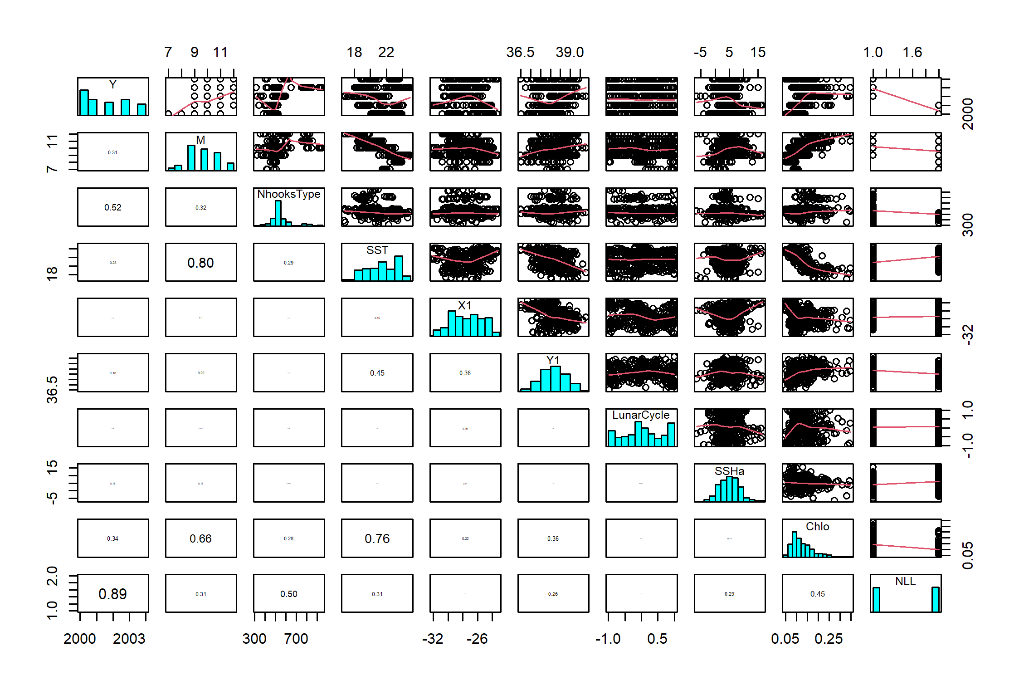


S1 Fig. Correlation analysis with all candidate variables to perform the generalized additive mixed model (GAMMs) analyses.

S3 Table. Summary steps of the backward model selection process, including the initial full model and final model for each species. The final models are highlighted in bold. ED = explained deviance, NS = non-significant variables

| **Species** | **Backward selection** | **Model** | **AIC** | **REML/UBRE** | **ED (%)** | **Family** | **NS** |
| --- | --- | --- | --- | --- | --- | --- | --- |
| Blue Shark | Full model | N.IND~HT+Y+s(SST)+s(SSHa)+s(LC, bs="cc")+s(X1)+(Y1)+offset(log(Effort))+s(Date, bs="re") | 5351.75 | 2982.50 | 96.30 | Negative Binomial | LC |
|  | Model 1 | N.IND~HT+Y+s(SST)+s(SSHa)+s(X1)+(Y1)+offsetlog(Effort))+s(Date, bs="re") | 5345.21 | 2982.70 | 96.30 | Negative Binomial | - |
|  | **Model 2** | **N.IND~HT+Y+s(SST)+s(SSHa)+s(X1,Y1)+offset(log(Effort))+s(Date, bs="re")** | 5339.18 | 2960.80 | 95.90 | Negative Binomial | - |
| Swordfish | Full model | N.IND~HT+Y+s(SST)+s(SSHa)+s(LC, bs="cc")+s(X1)+(Y1)+offsetlog(Effort))+s(Date, bs="re") | 3666.50 | 0.3408 | 50.80 | Poisson | SSHa and LC |
|  | **Model 1** | **N.IND~HT+Y+s(SST)+s(X1)+(Y1)+offset(log(Effort))+s(Date, bs="re")** | 3666.20 | 0.3404 | 50.80 | Poisson | - |
|  | Model 2 | N.IND~HT+Y+s(SST)+s(X1,Y1)+offset(log(Effort))+s(Date, bs="re") | 3668.30 | 0.3428 | 51.00 | Poisson | - |
| Loggerhead | Full model | N.IND~HT+Y+s(SST)+s(SSHa)+s(LC, bs="cc")+s(X1)+(Y1)+offset(log(Effort))+s(Date, bs="re") | 1356.21 | 665.37 | 70.90 | Zero Inflated Poisson | SSHa, FL, Y1 |
|  | Model 1 | N.IND~HT+Y+s(SST)+s(X1)+(Y1)+offset(LogNHOOK)+s(Date, bs="re") | 1341.68 | 663.97 | 71.10 | Zero Inflated Poisson | Y1 |
|  | **Model 2** | **N.IND~HT+s(SST)+s(X1,Y1)+offset(log(Effort))+s(Date, bs="re")** | 1338.80 | 663.97 | 71.10 | Zero Inflated Poisson | - |
| Shortfin Mako | Full model | N.IND~HT+Y+s(SST)+s(SSHa)+s(LC, bs="cc")+s(X1)+(Y1)+offset(log(Effort))+s(Date, bs="re") | 1340.56 | 663.95 | 58.70 | Zero Inflated Poisson | SSHa |
|  | **Model 1** | **N.IND~HT+Y+s(SST)+s(LC,bs="cc")+offset(log(Effort))+s(Date,bs="re")** | 1342.23 | 665.86 | 58.90 | Zero Inflated Poisson |  |
|  | Model 2 | N.IND~HT+Y+s(SST)+s(LC, bs="cc")+s(X1,Y1)+offset(log(Effort))+s(Date,bs="re") | 1350.08 | 665.60 | 59.20 | Zero Inflated Poisson |  |

**Diagnostics plots (final models)**

***Blue Shark***


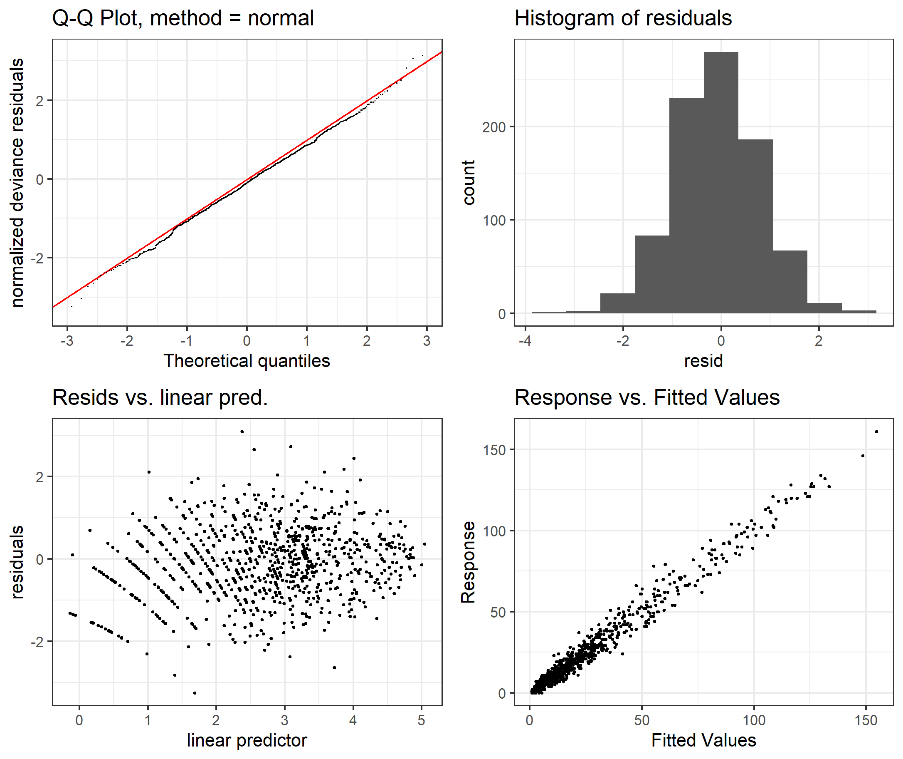


S2 Fig. Diagnostic plots of the final GAMM model for blue shark. Top left panel: normal QQ-plot of deviance residuals against theoretical quantiles; top right panel: histogram of the Pearson residuals; bottom left: residuals against the linear predictor; and bottom right: plot of observed vs fitted values.

***Swordfish***


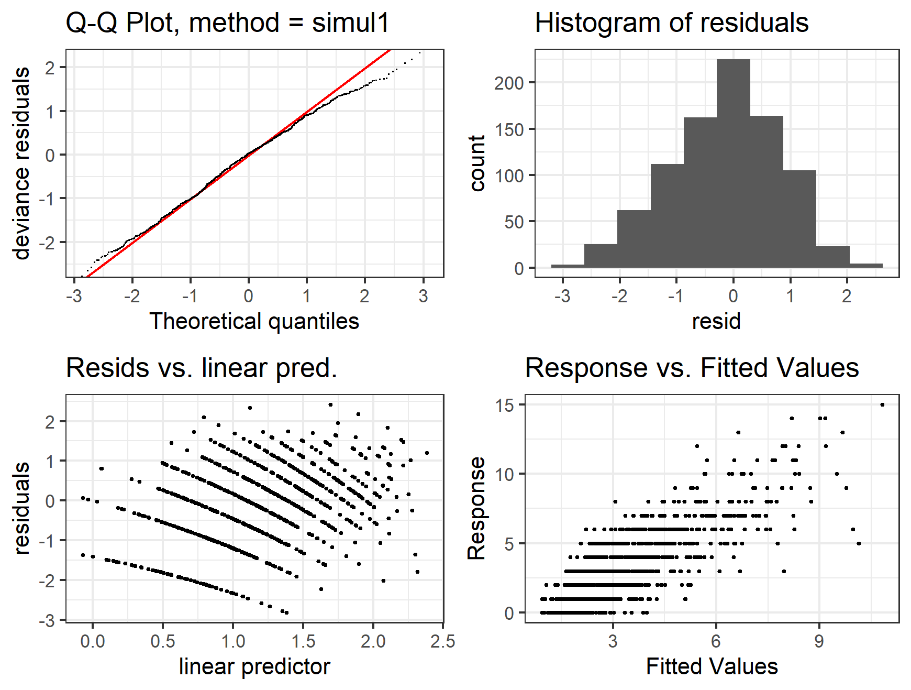


S3 Fig. Diagnostic plots of the final GAMM model for swordfish. Top left panel: normal QQ-plot of deviance residuals against theoretical quantiles; top right panel: histogram of the Pearson residuals; bottom left: residuals against the linear predictor; and bottom right: plot of observed vs fitted values.

***Loggerhead sea turtle***


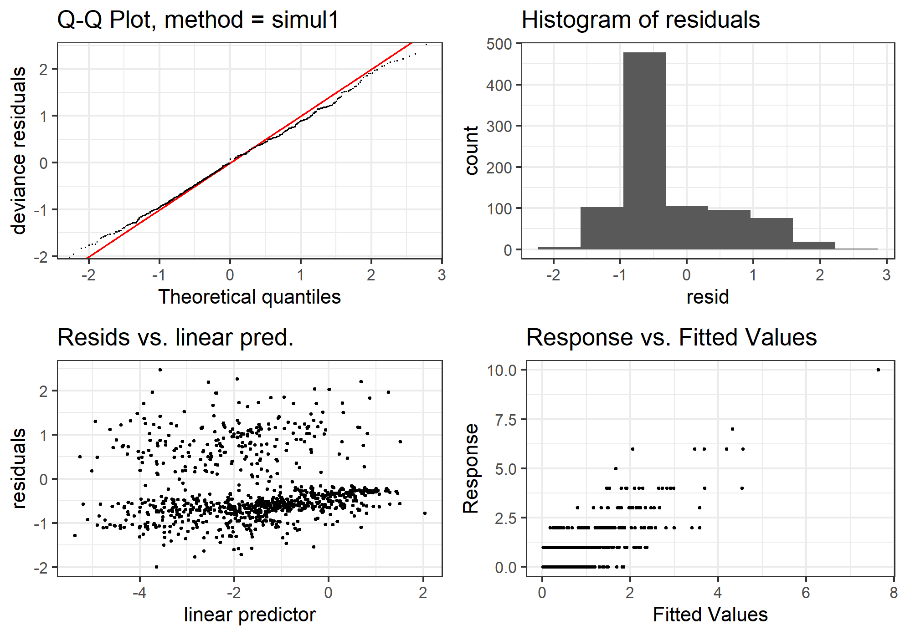


S4 Fig. Diagnostic plots of the final GAMM model for loggerhead sea turtle. Top left panel: normal QQ-plot of deviance residuals against theoretical quantiles; top right panel: histogram of the Pearson residuals; bottom left: residuals against the linear predictor; and bottom right: plot of observed vs fitted values.

***Shortfin mako***


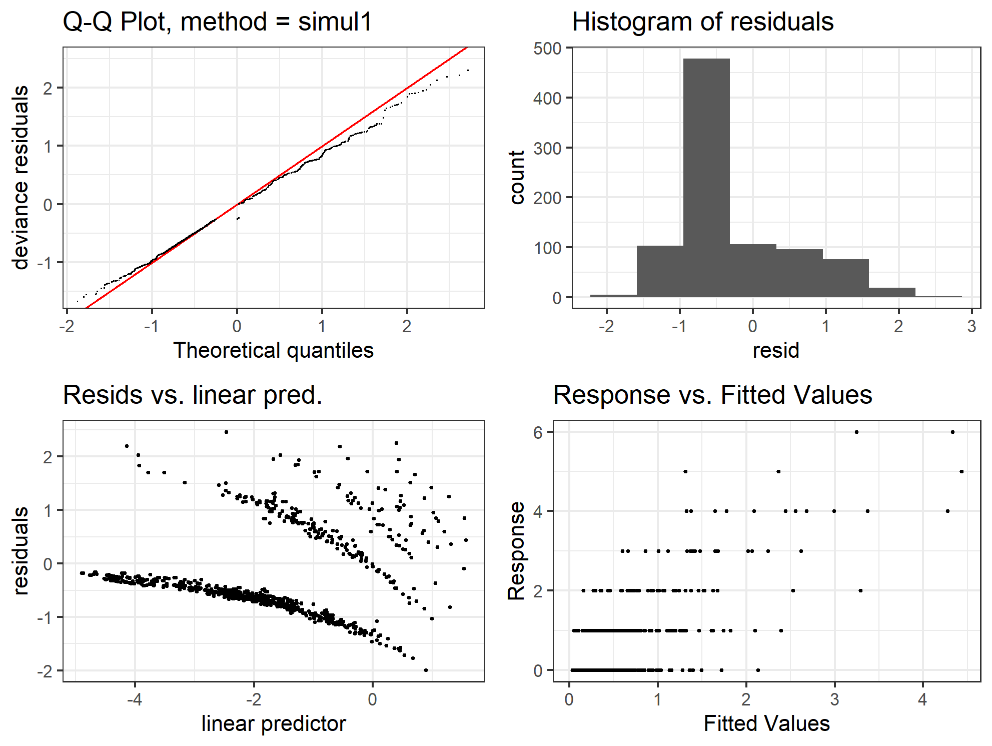


S5 Fig. Diagnostic plots of the final GAMM model for shortfin mako. Top left panel: normal QQ-plot of deviance residuals against theoretical quantiles; top right panel: histogram of the Pearson residuals; bottom left: residuals against the linear predictor; and bottom right: plot of observed vs fitted values.

**RELATIVE SIZE SELECTIVITY – BACKWARD MODEL SELECTION**

S4 Table. Summary steps of the backward model selection process, including the initial full model and final model for each species. The final models are highlighted in bold. ED = explained deviance, NS = non-significant variables

| **Species** | **Backward selection** | **Model** | **AIC** | **UBRE** | **ED (%)** | **NS** |
| --- | --- | --- | --- | --- | --- | --- |
| Blue Shark | Full model | PCL~HT+Y+s(SST)+s(SSHa)+s(LC, bs="cc")+s(X1)+s(Y1)+s(Date, bs="re") | 24575.52 | 0.1724 | 16.90 | SSHa, X1 |
|  | Model 1 | PCL~HT+Y+s(SST)+s(LC, bs="cc")+s(X1)+s(Y1)+s(Date, bs="re") | 24577.10 | 0.1725 | 16.90 | - |
|  | **Model 2** | **PCL~HT+Y+s(SST)+s(LC, bs="cc")+s(X1,Y1)+s(Date, bs="re")** | 24573.22 | 0.1723 | 17.00 | - |
| Swordfish | Full model | LJFL~HT+Y+s(SST)+s(SSHa)+s(LC, bs="cc")+s(X1)+s(Y1)+s(Date, bs="re") | 3233.67 | 0.3616 | 4.53 | SST, SSHa, X1, Y1, HT |
|  | Model 1 | LJFL~HT+Y+s(LC, bs="cc")+s(X1)+s(Y1)+s(Date, bs="re") | 3135.09 | 0.3200 | 3.10 | SSHa, X1, HT |
|  | Model 2 | LJFL~HT+Y+s(LC, bs="cc")+s(X1, Y1)+s(Date, bs="re") | 3233.13 | 0.3608 | 4.34 | (X1,Y1), HT |
|  | **Model 3*** | **LJFL~HT+s(LC, bs="cc")+s(X1)+s(Date, bs="re")** | 3255.77 | 0.3708 | 4.74 | - |
| Loggerhead | Full model | CCL~HT+Y+s(SST)+s(SSHa)+s(LC, bs="cc")+s(X1)+s(Y1)+s(Date, bs="re") | 499.64 | 0.1253 | 24.90 | Y, SSHa, Y1, LC, HT |
|  | Model 1 | CCL~HT+s(SST)+s(X1)+s(Date, bs="re") | 535.77 | 0.2067 | 24.60 | SST |
|  | **Model 2** | **CCL~HT+s(X1)+s(Date, bs="re")** | 537.90 | 0.2115 | 25.00 | - |
| Shortfin Mako | Full model | PCL~HT+Y+s(SST)+s(SSHa)+s(LC, bs="cc")+s(X1)+(Y1)+s(Date, bs="re") | 453.17 | 0.2520 | 26.10 | Y, SSHa, LC, HT |
|  | Model 1 | PCL~HT+s(SST)+s(X1)+(Y1)+s(Date, bs="re") | 466.74 | 0.2893 | 26.60 | SST, X1, Y1 |
|  | **Model 2** | **PCL~HT+s(Date, bs="re")** | 483.70 | 0.3180 | 21.40 | - |

**Correlation and Diagnostics plots (final binomial models)**

***Blue shark***


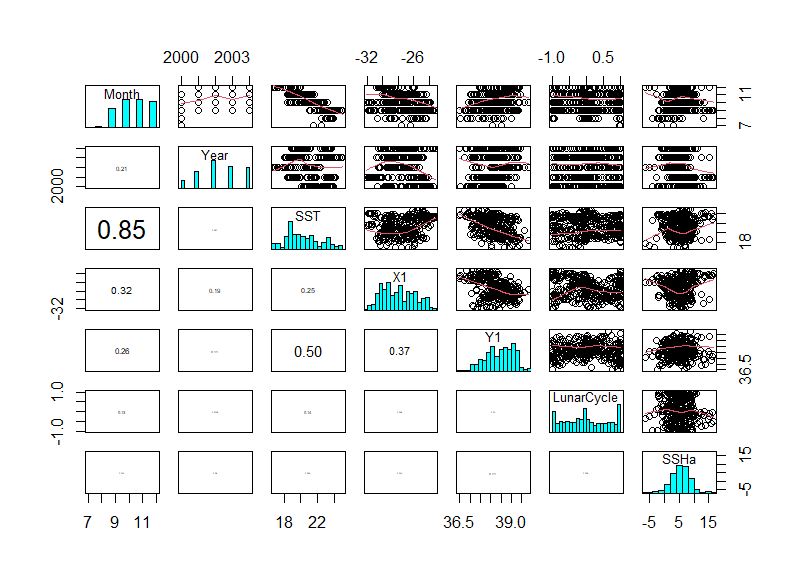


**A**


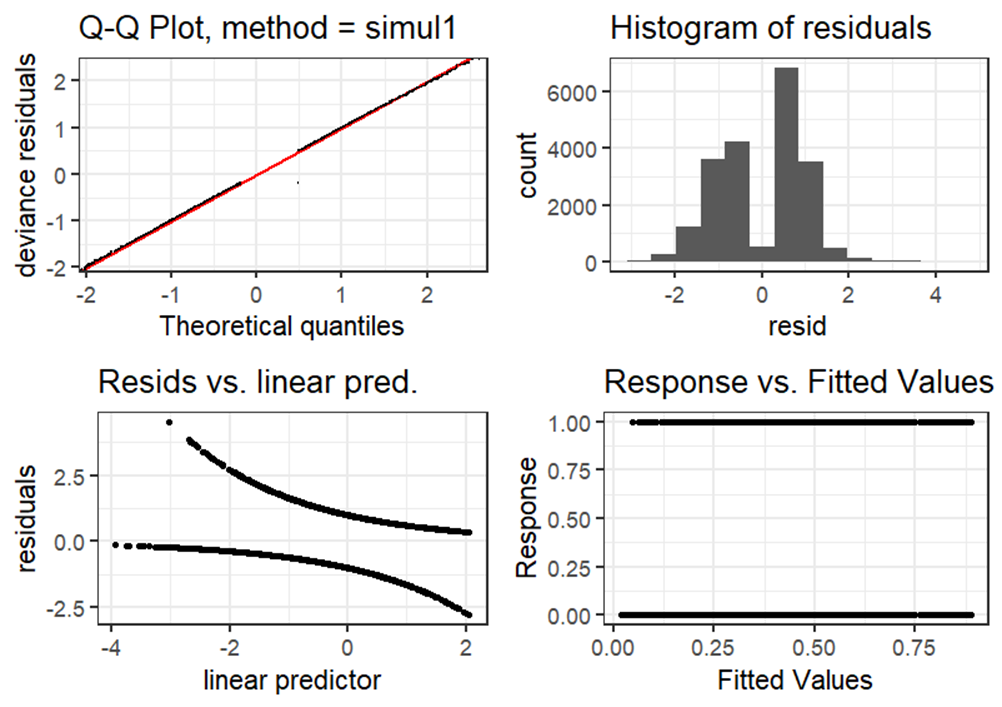


**B**

S6 Fig. A - Correlation analysis with all candidate variables to perform the generalized additive mixed model (GAMMs) analyses. B - Diagnostic plots of the final GAMM binomial model for blue shark. Top left panel: normal QQ-plot of deviance residuals against theoretical quantiles; top right panel: histogram of the Pearson residuals; bottom left: residuals against the linear predictor; and bottom right: plot of observed vs fitted values.

***Swordfish***

***
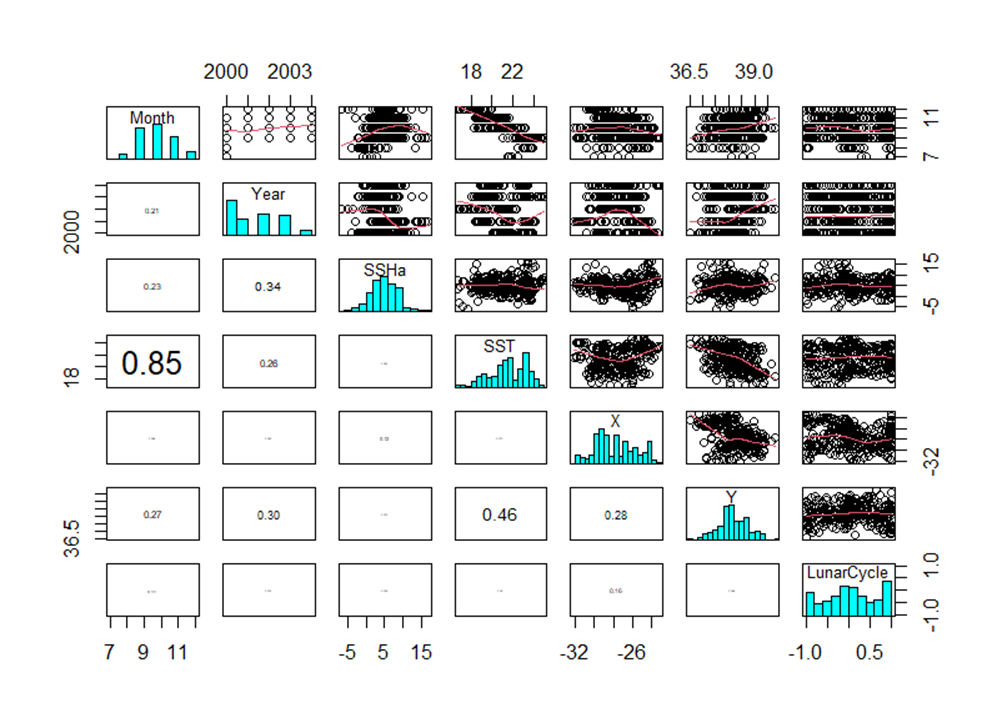
***

**A**

**B**


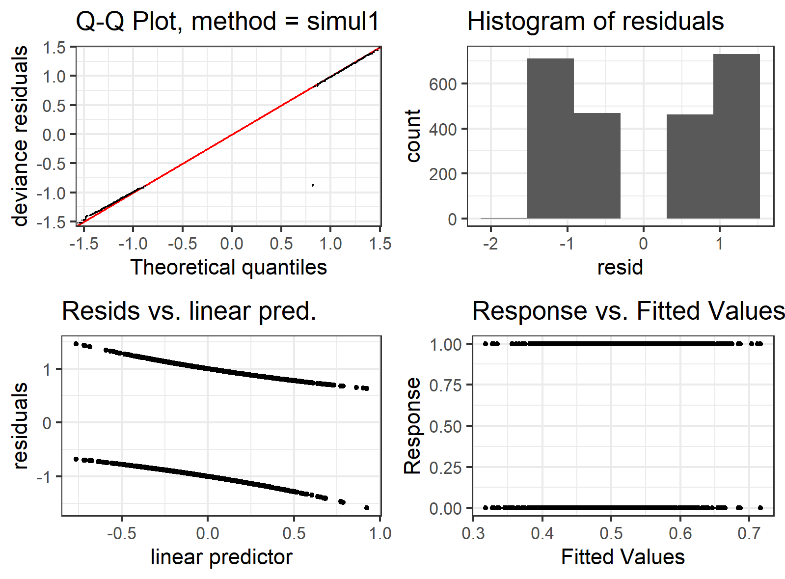


S7 Fig. A - Correlation analysis with all candidate variables to perform the generalized additive mixed model (GAMMs) analyses. B - Diagnostic plots of the final GAMM binomial model for swordfish. Top left panel: normal QQ-plot of deviance residuals against theoretical quantiles; top right panel: histogram of the Pearson residuals; bottom left: residuals against the linear predictor; and bottom right: plot of observed vs fitted values.

***Loggerhead sea turtle***

***
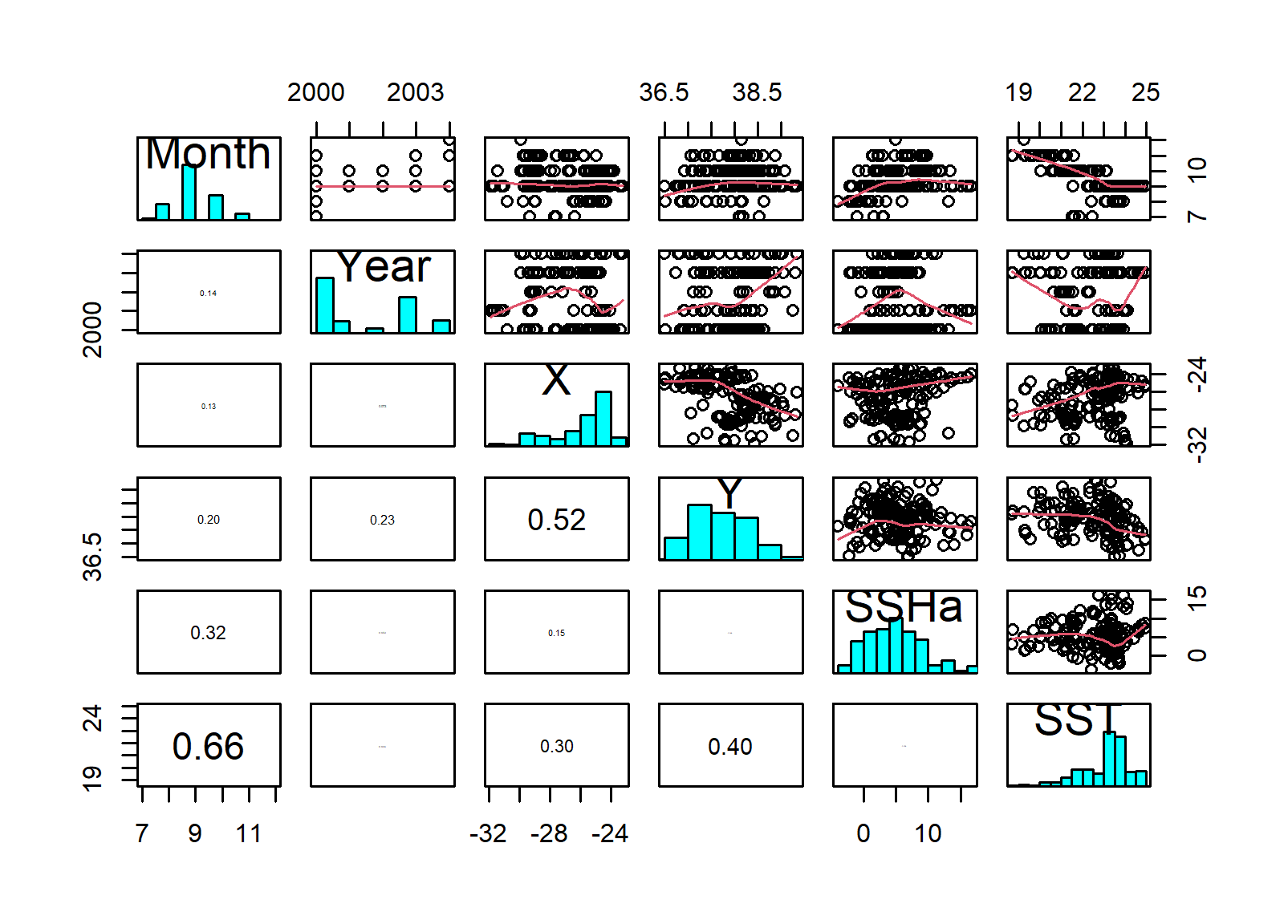
***

**A**


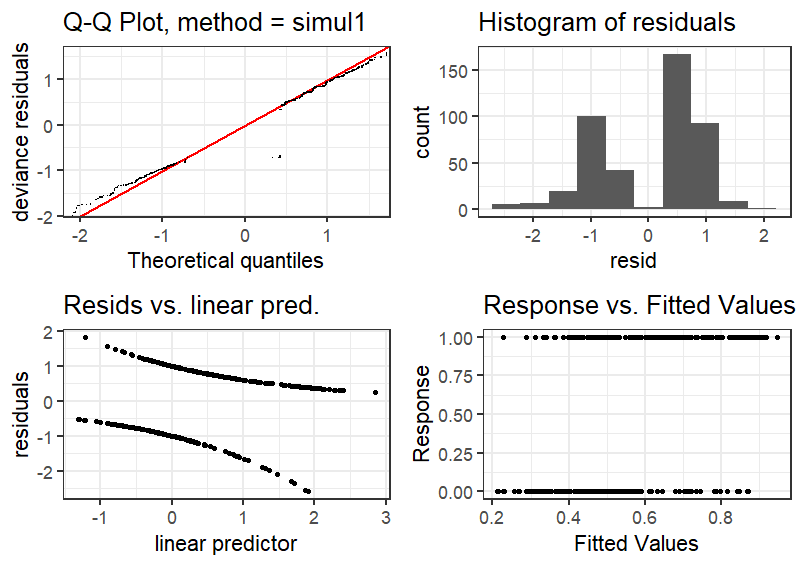


**B**

S8 Fig. A - Correlation analysis with all candidate variables to perform the generalized additive mixed model (GAMMs) analyses. B - Diagnostic plots of the final GAMM binomial model for loggerhead sea turtle. Top left panel: normal QQ-plot of deviance residuals against theoretical quantiles; top right panel: histogram of the Pearson residuals; bottom left: residuals against the linear predictor; and bottom right: plot of observed vs fitted values.

**Shortfin mako**

**
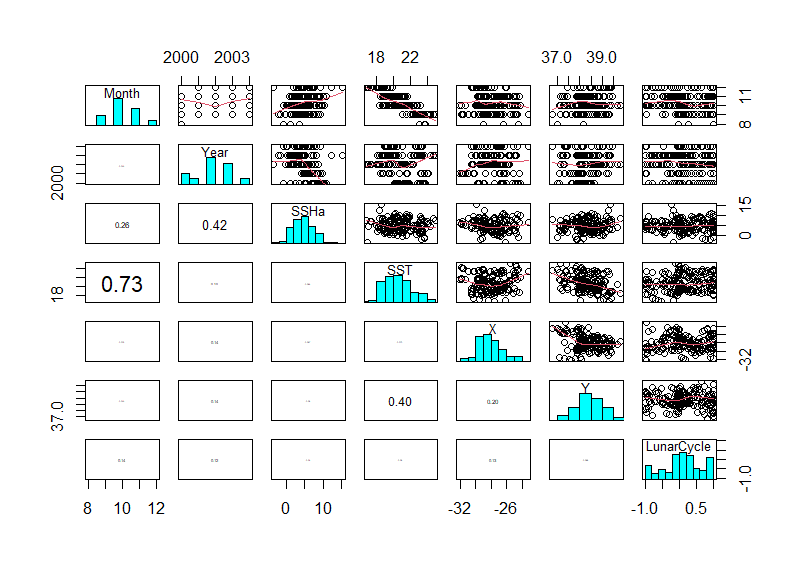
**

**A**

**B**


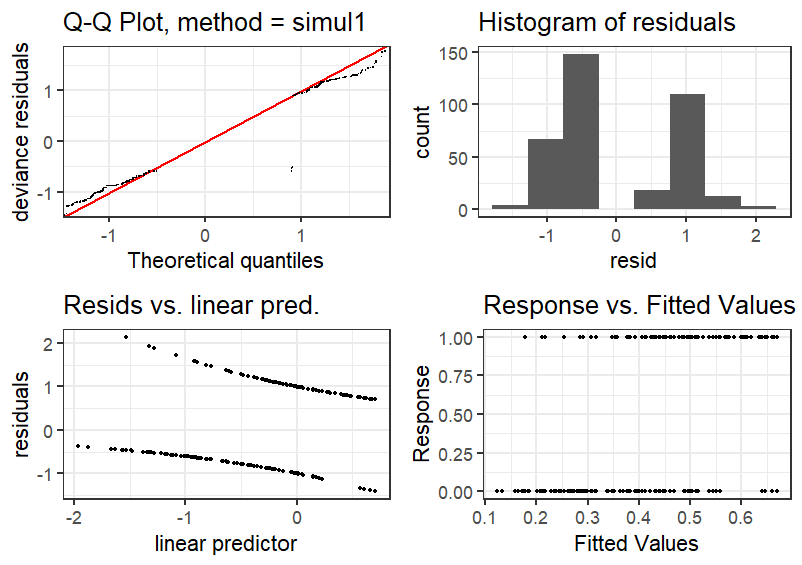


S9 Fig. A - Correlation analysis with all candidate variables to perform the generalized additive mixed model (GAMMs) analyses. B - Diagnostic plots of the final GAMM binomial model for shortfin mako. Top left panel: normal QQ-plot of deviance residuals against theoretical quantiles; top right panel: histogram of the Pearson residuals; bottom left: residuals against the linear predictor; and bottom right: plot of observed vs fitted values.
